# A model-free approach to do long-term volatility forecasting and its variants

**DOI:** 10.1186/s40854-023-00466-6

**Published:** 2023-03-01

**Authors:** Kejin Wu, Sayar Karmakar

**Affiliations:** 1 Department of Mathematics, University of California San Diego, La Jolla, USA; 2 Department of Statistics, University of Florida, Gainesville, USA

**Keywords:** ARCH-GARCH, Model free, Aggregated forecasting

## Abstract

Volatility forecasting is important in financial econometrics and is mainly based on the application of various GARCH-type models. However, it is difficult to choose a specific GARCH model that works uniformly well across datasets, and the traditional methods are unstable when dealing with highly volatile or short-sized datasets. The newly proposed normalizing and variance stabilizing (NoVaS) method is a more robust and accurate prediction technique that can help with such datasets. This model-free method was originally developed by taking advantage of an inverse transformation based on the frame of the ARCH model. In this study, we conduct extensive empirical and simulation analyses to investigate whether it provides higher-quality long-term volatility forecasting than standard GARCH models. Specifically, we found this advantage to be more prominent with short and volatile data. Next, we propose a variant of the NoVaS method that possesses a more complete form and generally outperforms the current state-of-the-art NoVaS method. The uniformly superior performance of NoVaS-type methods encourages their wide application in volatility forecasting. Our analyses also highlight the flexibility of the NoVaS idea that allows the exploration of other model structures to improve existing models or solve specific prediction problems.

## Introduction

In financial econometrics, forecasting volatility accurately and robustly is an important task (Engle and Patton 2001; Du and Budescu 2007). High-quality volatility forecasting is crucial for practitioners and traders to make decisions on risk management, asset allocation, price of the derivative instrument, and fiscal policies (Fang et al. 2018; Ashiya 2003; Bansal et al. 2016; Kitsul and Wright 2013; Morikawa 2019). However, volatility forecasting is challenging due to factors such as a small sample size, heteroscedasticity, and structural change (Chudý et al. 2020). Standard methods for volatility forecasting are typically built upon GARCH-type models; these models’ abilities to forecast the absolute magnitude and quantiles or the entire density of squared financial log-returns (i.e., equivalent to volatility forecasting to some extent)^1^ were shown by Engle and Patton (2001) using the Dow Jones Industrial Index. Later, many studies compared the performances of different GARCH-type models in volatility prediction; see Chortareas et al. (2011), González-Rivera et al. (2004), Herrera et al. (2018), Lim and Sek (2013), Peters (2001), Wilhelmsson (2006) and Zheng (2012). Some researchers attempted to develop the GARCH model further, such as by adopting smoothing parameters or adding more related information to estimate models (Breitung and Hafner 2016; Chen et al. 2012; Fiszeder and Perczak 2016; Taylor 2004). To model the proper process of volatility during the fluctuating period, Kim et al. (2011) applied time series models with stable and tempered-stable innovations to measure market risk during the highly volatile period, Ben Nasr et al. (2014) applied a fractionally integrated time-varying GARCH (FITVGARCH) model to fit volatility, and Karmakar and Roy (2021) developed a Bayesian method to estimate time-varying analogs of ARCH-type models to describe frequent volatility changes. Although there are several types of GARCH models, it is difficult to determine which one outperforms others uniformly because the performances of these models heavily depend on the error distribution, length of the prediction horizon, and property of the dataset.

To overcome this dilemma, we adhere to a recently developed model-free method, NoVaS, which applies normalizing and variance-stabilizing transformation (NoVaS transformation) to perform predictions. The NoVaS method is guided by the Model-free Prediction Principle, first proposed by Politis (2003). Previous studies showed that the NoVaS method performs better than GARCH-type models in forecasting squared log-returns. Notably, Gulay and Emec (2018) showed that the NoVaS method could beat GARCH-type models (GARCH, EGARCH, and GJR-GARCH) with generalized error distributions by comparing the pseudo-out-of-sample^2^(POOS) forecasting performance. Furthermore, Chen and Politis (2019) found an approach to perform multi-step-ahead predictions of squared log-returns based on the NoVaS method. Wu and Karmakar (2021) further substantiated the effective performance of NoVaS methods on aggregated long-term (30-steps ahead) predictions. In a recent study, Wang and Politis (2022) applied a model-free idea to provide estimation and prediction inferences for a general class of time series. Although they adopted a two-stage transformation approach to achieve the model-free goal, which is different from the NoVaS method, the validity of such a Model-free Prediction Principle was shown. From a practical aspect of forecasting volatility, to obtain some inference about the future situation at an overall level, we choose the time-aggregated prediction metric taken by Wu and Karmakar (2021) to measure the short- and long-term forecasting performance of different methods. This aggregated metric has been applied to depict the future situation of electricity prices and financial data (Chudý et al. 2020; Karmakar et al. 2022; Fryzlewicz et al. 2008).

One drawback of the existing NoVaS-type methods is that the parameters of the transformation must obey a specific form, which decreases its flexibility. Inspired by the development of the ARCH model (Engle 1982) to the GARCH model (Bollerslev 1986), this study attempts to build a novel NoVaS method derived by iterating the GARCH(1,1) structure. Our new method provides more freedom in the region of the parameters. Moreover, to achieve a fair and comprehensive comparison between NoVaS-type and standard GARCH methods, we simulated data from various models to examine their robustness. On the empirical side, we split volatility forecasting into three main classes, that is, considering the volatility prediction of stock, currency, and index data. Through extensive data analyses, we show that all NoVaS-type methods bring significant improvements compared with the standard GARCH model when the available data are short or volatile. Moreover, our new methods generally perform better than current NoVaS methods.

The remainder of this paper is organized as follows. Details about the existing NoVaS method and the motivations for proposing our new method are explained in “NoVaS method and evaluation metric” section. We also explain the evaluation metrics used throughout this study. In “New variants of the NoVaS method” section, we propose a new NoVaS transformation approach and its parsimonious variants. To compare all NoVaS-type methods with the standard GARCH model, POOS predictions on simulated and real-world datasets were performed using “Simulation” and “Real-world data analysis” Sections. In “Comparison of predictive accuracy: Statistical tests” section, we present statistical test results to substantiate our new methods. Finally, the discussion and conclusion are presented in “Results and discussion” and “Conclusion” sections, respectively.

## NoVaS method and evaluation metric

In this section, we first introduce the Model-free Prediction Principle. We then present how the NoVaS transformation can be built from an ARCH model. Subsequently, the motivation to build a new NoVaS transformation and time-aggregated metric is provided.

### Model-free prediction principle

Before presenting the NoVaS method in detail, we throw some light on the insight of the Model-free Prediction Principle. The main idea behind this is to apply an invertible transformation function $H_T$ that can map a non-*i*.*i*.*d*. vector $\{Y_t~;t = 1,\ldots ,T\}$ to a vector $\{\epsilon _t;~t=1,\ldots ,T\}$ with *i*.*i*.*d*. components. Because the prediction of *i*.*i*.*d*. data is somewhat standard, the prediction of $Y_{T+1}$ can easily be obtained by inversely transforming ${\hat{\epsilon }}_{T+1}$ which is a prediction of $\epsilon _{T+1}$ using $H_T^{-1}$. In other words, we can express prediction ${\hat{Y}}_{T+1}$ as a function of $\varvec{Y}_T$, $\varvec{X}_{T+1}$ and ${\hat{\epsilon }}_{T+1}$:

1 $$\begin{aligned} {\hat{Y}}_{T+1}=f_{T+1}(\varvec{Y}_{T}, \varvec{X}_{T+1},{\hat{\epsilon }}_{T+1}), \end{aligned}$$

where $\varvec{Y}_{T}$ denotes all historical data $\{Y_t;~t =1,\ldots ,T\}$, $\varvec{X}_{T+1}$ is the collection of all predictors, and it also contains the value of a future predictor $X_{T+1}$. In this article, we show how to build NoVaS transformations related to ARCH and GARCH models. After acquiring Eq. (1), we can even predict $g(Y_{T+1})$, where $g(\cdot )$ is a general continuous function. Politis (2015) defined two data-based optimal predictors of $g(Y_{T+1})$ under $L_1$ (Mean Absolute Deviation) and $L_2$ (Mean Squared Error) loss criteria respectively as below:

2 $$\begin{aligned} \begin{aligned} g(Y_{T+1})_{L_2}&= \frac{1}{M}\sum _{m=1}^Mg(f_{T+1}(\varvec{Y}_T,\varvec{X}_{T+1},{\hat{\epsilon }}_{T+1,m})),\\ g(Y_{T+1})_{L_1}&= \text {Median of }\{g(f_{T+1}(\varvec{Y}_T,\varvec{X}_{T+1},{\hat{\epsilon }}_{T+1,m}));m = 1,\ldots ,M\}. \end{aligned} \end{aligned}$$

In Eq. (2), $\{{\hat{\epsilon }}_{T+1,m}\}_{m=1}^{M}$ are generated by Bootstrap or Monte Carlo method; see more details in “GE-NoVaS method” section; *M* takes a large number of 5000 in this study.

### NoVaS transformation

The NoVaS transformation is a straightforward application of the Model-free Prediction Principle,^3^ which is based on the ARCH model introduced by Engle (1982), as follows:

3 $$\begin{aligned} Y_t = W_t\sqrt{a+\sum _{i=1}^pa_iY_{t-i}^2}. \end{aligned}$$

In Eq. (3), these parameters satisfy $a\ge 0$ and $a_i\ge 0$ for all $i = 1,\ldots ,p$ and $W_t\sim i.i.d.~N(0,1)$. In other words, the structure of the ARCH model provides a ready-made $H_T$. We express $W_t$ in Eq. (3) using the following terms:

4 $$\begin{aligned} W_t = \frac{Y_t}{\sqrt{a+\sum _{i=1}^pa_iY_{t-i}^2}} ~;~\text {for}~ t=p+1,\ldots ,T. \end{aligned}$$

Subsequently, Eq. (4) can be considered a potential form of $H_T$. Additional adjustments were performed by Politis (2003) to obtain the modified Eq. (5):

5 $$\begin{aligned} W_{t}=\frac{Y_t}{\sqrt{\alpha s_{t-1}^2+\beta Y_t^2+\sum _{i=1}^pa_iY_{t-i}^2}}~;~\text {for}~ t=p+1,\ldots ,T. \end{aligned}$$

In Eq. (5), $\{Y_t;~t=1,\ldots ,T\}$ are the target data, such as financial log-returns in this study; $\{W_{t};~t=p+1,\ldots ,T\}$ is the transformed vector; $\alpha$ is a fixed scale invariant constant; $s_{t-1}^2$ is an estimator of the variance of $\{Y_i;~i = 1,\ldots ,t-1\}$ and can be calculated by $(t-1)^{-1}\sum _{i=1}^{t-1}(Y_i-\mu )^2$, where $\mu$ is the mean of $\{Y_i;~i = 1,\ldots ,t-1\}$. $\{W_t\}_{t = p+1}^{T}$ expressed in Eq. (5) are assumed to be *i*.*i*.*d*. *N*(0, 1); however, this is not the case. To make Eq. (5) a qualified function $H_T$, that is, making $\{W_t\}_{t=p+1}^{T}$ obey the standard normal distribution, we still need to impose some restrictions on $\alpha$ and $\beta , a_1,\ldots ,a_p$. Hence, first, we stabilize the variance by requiring

6 $$\begin{aligned} \alpha \ge 0, \beta \ge 0, a_i\ge 0~;~\text {for all}~i\ge 1, \alpha + \beta + \sum _{i=1}^pa_i=1. \end{aligned}$$

By imposing the above requirement, we can make $\{W_t\}_{t=p+1}^{T}$ series possess approximate unit variance. Importantly, we must also make $\{W_t\}_{t=p+1}^{T}$ independent. In practice, $\{W_t\}_{t=p+1}^{T}$ transformed from financial log-returns by the NoVaS transformation are usually uncorrelated.^4^ Therefore, if we make $\{W_t\}_{t=p+1}^{T}$ close to a Gaussian series, that is, normalizing $\{W_t\}_{t=p+1}^{T}$, we can obtain the desired *i*.*i*.*d*. transformed series. Note that the distribution of financial log-returns is usually symmetric; thus, kurtosis can serve as a simple distance to measure the departure of a non-skewed dataset from that of the standard normal distribution (Politis 2015). In addition, matching the marginal distribution seems sufficient to normalize the joint distribution of $\{W_t\}_{t=p+1}^{T}$ for practical purposes, based on empirical results. We use ${\hat{F}}_w$ to denote the marginal distribution of $\{W_t\}_{t=p+1}^{T}$ and use $KURT(W_t)$ to denote the kurtosis of ${\hat{F}}_w$. Thus, to bring ${\hat{F}}_w$ close to the standard normal distribution, we attempt to minimize $|KURT(W_t)-3|$^5^ to obtain an optimal combination of $\alpha ,\beta ,a_1,\ldots ,a_p$. Consequently, the NoVaS transformation was determined.

According to Chen (2018), based on the prediction accuracy and model structure, the Generalized Exponential NoVaS (GE-NoVaS) method is the most reasonable among the different NoVaS-type methods with an exponentially decayed form of $\{a_i\}_{i=1}^p$:

7 $$\begin{aligned} \alpha \ne 0, \beta = c', a_i = c'e^{-ci}~;~\text {for all}~1\le i\le p, c' = \frac{1-\alpha }{\sum _{j=0}^pe^{-cj}}. \end{aligned}$$

In this study, we verified the advantages of our new methods by comparing them with the GE-NoVaS method. Before further proposing the new NoVaS transformation, we discuss in more detail the GE-NoVaS method and our motivations for creating new methods.

### GE-NoVaS method

For the GE-NoVaS method, the fixed $\alpha$ is larger than 0 and selected from a grid of possible values based on prediction performance. In this study, we define this grid as $(0.1,0.2,\ldots ,0.8)$, containing eight discrete values.^6^ From Eq. (2.2), using the Model-free Prediction Principle, we can obtain the function $H_T$ of the GE-NoVaS method by requiring the parameters to satisfy Eq. (7) and minimizing $|KURT(W_t)-3|$. To complete the model-free prediction process, we must still determine the form of $H_T^{-1}$. From Eq. (5), $H_T^{-1}$ can be written as follows:

8 $$\begin{aligned} Y_t=\sqrt{\frac{W_{t}^2}{1-\beta W_{t}^2}(\alpha s_{t-1}^2+\sum _{i=1}^pa_iY_{t-i}^2)}~;~\text {for}~ t=p+1,\ldots ,T. \end{aligned}$$

We can easily obtain the analytical form of $Y_{T+1}$, which can be expressed as

9 $$\begin{aligned} Y_{T+1}=\sqrt{\frac{W_{T+1}^2}{1-\beta W_{T+1}^2}(\alpha s_{T}^2+\sum _{i=1}^pa_iY_{T+1-i}^2)}. \end{aligned}$$

In Eq. (9), $s_T^2$ is an estimator of the variance of $\{Y_t;~t=1,\ldots ,T\}$ and can be calculated using $T^{-1}\sum _{i=1}^T(Y_i-\mu )^2$, $\mu$ is the mean of the data. Based on Eq. (2), we can define $L_1$ and $L_2$ optimal predictors of $Y_{T+1}^2$ after observing the historical information set ${\mathscr {F}}_{T} = \{Y_t,1\le t \le T\}$ as follow:

10 $$\begin{aligned} \begin{aligned} L_1\text {-optimal predictor of}~&Y_{T+1}^2:\\&\text {Median}\left\{ Y_{T+1,m}^2; m=1,\ldots ,M\big |{\mathscr {F}}_{T}\right\} \\&= \text {Median}\left\{ \frac{W_{T+1,m}^2}{1-\beta W_{T+1,m}^2}(\alpha s_{T}^2+\sum _{i=1}^pa_iY_{T+1-i}^2); m=1,\ldots ,M \bigg |{\mathscr {F}}_{T}\right\} \\&=(\alpha s_{T}^2+\sum _{i=1}^pa_iY_{T+1-i}^2)\text {Median}\left\{ \frac{W_{T+1,m}^2}{1-\beta W_{T+1,m}^2}; m=1,\ldots ,M\right\} ,\\ L_2\text {-optimal predictor of}~&Y_{T+1}^2:\\&\text {Mean}\left\{ Y_{T+1,m}^2; m=1,\ldots ,M \big |{\mathscr {F}}_{T}\right\} \\&= \text {Mean}\left\{ \frac{W_{T+1,m}^2}{1-\beta W_{T+1,m}^2}(\alpha s_{T}^2+\sum _{i=1}^pa_iY_{T+1-i}^2); m=1,\ldots ,M\bigg |{\mathscr {F}}_{T}\right\} \\&=(\alpha s_{T}^2+\sum _{i=1}^pa_iY_{T+1-i}^2)\text {Mean}\left\{ \frac{W_{T+1,m}^2}{1-\beta W_{T+1,m}^2}; m=1,\ldots ,M\right\} , \end{aligned} \end{aligned}$$

where $\{W_{T+1,m}\}_{m=1}^{M}$ is bootstrapped *M* times from their empirical distribution or generated from a trimmed standard normal distribution^7^ by using the Monte Carlo method. That is, $Y_{T+1}$ can be represented as a function of $W_{T+1}$ and ${\mathscr {F}}_{T}$ as follows:

11 $$\begin{aligned} Y_{T+1} = f_{GE}(W_{T+1};{\mathscr {F}}_{T}). \end{aligned}$$

To remind us of the relationship between $Y_{T+1}$ and $W_{T+1}, Y_1, \ldots , Y_T$ derived from the GE-NoVaS method, we use $f_{GE}(\cdot )$ to denote this function. It is not difficult to determine that $Y_{T+2}$ can be expressed as

12 $$\begin{aligned} \begin{aligned} Y_{T+2}&= \sqrt{\frac{W_{T+2}^2}{1-\beta W_{T+2}^2}(\alpha s_{T}^2+\sum _{i=1}^pa_iY_{T+2-i}^2)}\\&=f_{GE}(W_{T+1},W_{T+2};{\mathscr {F}}_{T}). \end{aligned} \end{aligned}$$

We can generate $\{W_{T+1,m},W_{T+2,m}\}_{m=1}^{M}$ *M* times to compute the $L_1$ and $L_2$ optimal predictors of $Y_{T+2}^{2}$ as we did for the 1-step ahead optimal prediction. Similarly, with $\{W_{T+1,m},\ldots ,$ $W_{T+h,m}\}_{m=1}^{M}$, we can accomplish the multi-step ahead optimal prediction of $Y_{T+h}^{2}$ for any $h\ge 3$. In summary, we can express $Y_{T+h}$ as

13 $$\begin{aligned} Y_{T+h} = f_{GE}(W_{T+1},\ldots ,W_{T+h};{\mathscr {F}}_{T})~;~\text {for any}~h\ge 1. \end{aligned}$$

Note that the analytical form of $Y_{T+h}$ from the GE-NoVaS transformation depends only on $i.i.d.~\{W_{T+1},\ldots ,W_{T+h}\}$ and ${\mathscr {F}}_{T}$.

### Motivations of building a new NoVaS transformation

*Structured form of coefficients* The current GE-NoVaS method simply sets $\beta , a_1,\ldots ,a_p$ to be exponentially decayed. This allows us to propose the following idea. Can we build a more rigorous form of $\beta , a_1,\ldots ,a_p$ based on the relevant model itself without assigning any prior form to the coefficients? In this study, a new approach to exploring the form of $\beta , a_1,\ldots ,a_p$ based on the GARCH(1,1) model is proposed. Subsequently, the GARCH-NoVaS (GA-NoVaS) transformation was built. This is discussed in “GA-NoVaS transformation” section.

*Changing the NoVaS transformation* Wu and Karmakar (2021) showed that the current state-of-the-art GE-NoVaS method still renders extremely large time-aggregated multi-step ahead predictions under $L_2$ risk measure sometimes. The reason for this phenomenon is that the denominator of Eq. (9) is small when the generated $W_t^*$ is very close to $\sqrt{1/\beta }$. In this situation, the prediction error is amplified. Moreover, when a long-step-ahead prediction is desired, this amplification will accumulate, and the final prediction will be ruined. Thus, a $\beta$-removing technique was applied to the GE-NoVaS method to obtain a GE-NoVaS-without-$\beta$ method. This is a parsimonious version of the GE-NoVaS method. Henceforth, we call this method the P-GE-NoVaS. Similarly, we can obtain a parsimonious variant of the GA-NoVaS method (P-GA-NoVaS) by reusing this technique. A discussion of these parsimonious methods is presented in “Parsimonious variant of the GA-NoVaS method” and “Connection of two parsimonious methods” sections.

### Long-term forecasting evaluation metric

We first describe how log-returns can be calculated from the following equation:

14 $$\begin{aligned} Y_t = 100\times \log (X_{t+1}/X_t) ~;~\text {for}~ t = 1,\ldots ,499~\text {or}~t = 1,\ldots ,249, \end{aligned}$$

where $\{X_t\}_{t = 1}^{250}$ and $\{X_t\}_{t = 1}^{500}$ are 1-year and 2-year price series, respectively. Next, we define the time-aggregated predictions of squared log-returns with three different lengths of the prediction horizon as

15 $$\begin{aligned} \begin{aligned} {\bar{Y}}_{k,1}^2&= {\hat{Y}}_{k+1}^2,~k=250,\ldots ,498 ~\text {or}~k=100,\ldots ,248,\\ {\bar{Y}}_{i,5}^2&= \frac{1}{5}\sum _{m=1}^5{\hat{Y}}^2_{i+m},~i = 250,\ldots ,494~\text {or}~i=100,\ldots ,244,\\ {\bar{Y}}_{j,30}^2&= \frac{1}{30}\sum _{m=1}^{30}{\hat{Y}}^2_{j+m},~j = 250,\ldots ,469~\text {or}~j=100,\ldots ,219. \end{aligned} \end{aligned}$$

In Eq. (15), ${\hat{Y}}_{k+1}^2,{\hat{Y}}_{i+m}^2,{\hat{Y}}_{j+m}^2$ are single-point predictions of realized squared log-returns by NoVaS-type methods or the benchmark method; ${\bar{Y}}_{k,1}^2$, ${\bar{Y}}_{i,5}^2$ and ${\bar{Y}}_{j,30}^2$ represent 1-step, 5-steps and 30-steps ahead aggregated predictions, respectively. More specifically, for exhausting the information contained in the dataset, we roll the 250 data points window through the whole dataset, that is, we use $\{Y_1,\ldots ,Y_{250}\}$ to predict $Y_{251}^2,\{Y_{251}^2,\ldots ,Y_{255}^2\}$ and $\{Y_{251}^2,\ldots ,Y_{280}^2\}$; then we use $\{Y_2,\ldots ,Y_{251}\}$ to predict $Y_{252}^2,\{Y_{252}^2,\ldots ,Y_{256}^2\}$ and $\{Y_{252}^2,\ldots ,Y_{281}^2\}$, for 1-step, 5-steps, and 30-steps aggregated predictions respectively, and so on. To explore the performance of three different prediction lengths with small data size, we roll the 100 data point window through the entire dataset. For example, with a prediction horizon of 30, we perform time-aggregated predictions on a large dataset 220 times.

To measure the forecasting performance of the different methods, we propose a time-aggregated metric based on Eq. (16).

16 $$\begin{aligned} P = \sum _{l}({\bar{Y}}_{l,h}^2-\sum _{m=1}^h(Y_{l+m}^2/h))^2~;~l \in \{k,i,j\}. \end{aligned}$$

In Eq. (16), setting $l = k,i,j$ means we consider 1-step, 5-steps, and 30-steps ahead time-aggregated predictions, respectively; ${\bar{Y}}_{l,h}^2$ is the *h*-step ($h\in \{1,5,30\}$) ahead time-aggregated volatility prediction, defined in Eq. (15); $\sum _{m=1}^h(Y_{l+m}^2/h)$ is the corresponding true aggregated value calculated from the realized squared log-returns. To compare various NoVaS-type methods with the traditional method, we set a benchmark method to fit one GARCH(1,1) model directly (GARCH-direct). In “Simulation” and “Real-world data analysis” sections, we applied this metric to the simulation and real data analyses. In addition, in “Comparison of predictive accuracy: statistical tests” section, statistical tests are deployed to explore the predictive accuracy of NoVaS methods further.

## New variants of the NoVaS method

In this section, we first propose the GA-NoVaS method which is directly developed from the GARCH(1,1) model without assigning any specific form of $\beta , a_1,\ldots ,a_p$. Then, the P-GA-NoVaS method is introduced by applying the $\beta$-removing technique. We also provide algorithms for these two new methods at the end.

### GA-NoVaS transformation

Recall that the GE-NoVaS method mentioned in “GE-NoVaS method” section, was built by exploiting the ARCH(*p*) model for a large *p*. Although the ARCH model is the basis of the GE-NoVaS method, the free parameters of the GE-NoVaS method are only *c* and $\alpha$. To represent $p+1$ number of coefficients using only two free parameters, some specific forms are assigned to $\beta , a_1,\ldots ,a_p$. Here, we attempt to use a more convincing approach to find $\beta , a_1,\ldots ,a_p$ directly, without assigning any prior form to these parameters. We call this NoVaS transformation method the GA-NoVaS.

The idea behind this new method was inspired by the fact that the GARCH(1,1) model is equivalent to the corresponding ARCH($\infty$) model. If we want to build a NoVaS transformation based on the GARCH(1,1) model, the denominator on the right-hand side of Eq. (4) should be replaced by the structure of the GARCH(1,1) model, which has the form Eq. (17):

17 $$\begin{aligned} \begin{aligned} Y_t&= \sigma _tW_t,\\ \sigma _t^2&=a + a_1Y_{t-1}^2 + b_1\sigma _{t-1}^2. \end{aligned} \end{aligned}$$

In Eq. (17), $a \ge 0$, $a_1 > 0$, $b_1 > 0$, and $W_t\sim i.i.d.~N(0,1)$. In other words, a potentially qualified transformation related to the GARCH(1,1) or ARCH($\infty$) model can be expressed as:

18 $$\begin{aligned} W_t = \frac{Y_t}{\sqrt{a + a_1Y_{t-1}^2 + b_1\sigma _{t-1}^2}}. \end{aligned}$$

However, recall that the core insight of the NoVaS method connects the original data with the transformed data using a qualified transformation function. A primary problem here is that the right-hand side of Eq. (18) contains terms other than $\{Y_t\}$. Thus, additional manipulations are required to build the GA-NoVaS method. In fact, we can finally derive the transformation functions $H_{T}$ and $H_{T}^{-1}$ corresponding to the GA-NoVaS method as follows:

19 $$\begin{aligned} W_t = \frac{Y_t}{\sqrt{ c_0Y_t^2+ \alpha s_{t-1}^2 + \sum _{i = 1}^{q}c_iY_{t-i}^2 }}~;~ Y_t = \sqrt{\frac{W_t^2}{1-c_0W_t^2}(\alpha s_{t-1}^2+\sum _{i=1}^qc_iY_{t-i}^2)}, \end{aligned}$$

where $t=q+1,\ldots ,T$; see “Appendix 1” for details of this deduction process and the form of $\{c_i\}_{i = 0}^{q}$.

#### Remark 1

(The difference between GA-NoVaS and GE-NoVaS methods) Compared with the existing GE-NoVaS method, the GA-NoVaS method possesses a completely different transformation structure. All coefficients except for $\alpha$ implied by the GE-NoVaS method are expressed as $\beta = c', a_i = c'e^{-ci}~$ $\text {for all}~1\le$ $i\le p$, $c' = \frac{1-\alpha }{\sum _{j=0}^pe^{-cj}}$. There are only two free parameters, *c* and $\alpha$. However, there are four free parameters $\beta , a_1, b_1$ and $\alpha$ in Eq. (35). For example, the coefficient of $Y_t^2$ in the GE-NoVaS method is $(1-\alpha )/(\sum _{j=0}^pe^{-cj})$. By contrast, the corresponding coefficient in the GA-NoVaS structure is $\beta (1-\alpha )/(\beta +(1-b_1)\sum _{i=1}^{q}a_1b_1^{i-1})$. We can assume that the freedom of coefficients within the GA-NoVaS is larger than the freedom in the GE-NoVaS. Simultaneously, the structure of the GA-NoVaS method is built from the GARCH(1,1) model directly without imposing any prior assumptions on the coefficients. We believe this is the reason why our GA-NoVaS method shows a better prediction performance in “Simulation” and “Real-world data analysis” sections.

Next, it is not difficult to express $Y_{T+h}$ as a function of $W_{T+1},\ldots , W_{T+h}$ and ${\mathscr {F}}_{T}$ using the GA-NoVaS method, as we did in “GE-NoVaS method” section:

20 $$\begin{aligned} Y_{T+h} = f_{GA}(W_{T+1},\ldots ,W_{T+h};{\mathscr {F}}_{T})~;~\text {for any}~h\ge 1. \end{aligned}$$

Once the expression of $Y_{T+h}$ is determined, we can apply the same procedure with the GE-NoVaS method to obtain the optimal predictor of $Y_{T+h}$ under $L_1$ or $L_2$ risk criterion. To address $\alpha$, we adopt the same strategy used in the GE-NoVaS method. Note that the value of $\alpha$ is invariant during the optimization process once it is fixed as a specific value. More details regarding the algorithm of this new method can be found in “Algorithms of new methods” section.

### Parsimonious variant of the GA-NoVaS method

According to the $\beta$-removing concept, we can continue to propose the P-GA-NoVaS method, which is a parsimonious variant of the GA-NoVaS method. First, we present the P-GE-NoVaS method from Wu and Karmakar (2021).

21 $$\begin{aligned} W_{t}=\frac{Y_t}{\sqrt{\alpha s_{t-1}^2+\sum _{i=1}^pa_iY_{t-i}^2}}~;~Y_t=\sqrt{W_{t}^2(\alpha s_{t-1}^2+\sum _{i=1}^pa_iY_{t-i}^2)}~;~\text {for}~ t=p+1,\ldots ,T. \end{aligned}$$

Equation (21) still needs to satisfy the requirement of normalizing and variance-stabilizing transformation. Therefore, we restrict $\alpha + \sum _{i=1}^pa_i = 1$ and select the optimal combination of $\alpha , a_1,\ldots ,a_p$ by minimizing $|KURT(W_t)-3|$. Then, $Y_{T+1}$ can be expressed as Eq. (22):

22 $$\begin{aligned} Y_{T+1}=\sqrt{W_{T+1}^2(\alpha s_{T}^2+\sum _{i=1}^pa_iY_{T+1-i}^2)}. \end{aligned}$$

#### Remark 2

Even though we do not include the effect of $Y_T$ when we build $H_T$, the expression of $Y_{T+1}$ still contains the current value $Y_T$. This means that the P-GE-NoVaS method does not disobey the causal prediction rule.

Similarly, the P-GA-NoVaS can be represented by the following equation:

23 $$\begin{aligned} W_t = \frac{Y_t}{\sqrt{ \alpha s_{t-1}^2 + \sum _{i = 1}^{q}\tilde{c}_iY_{t-i}^2 }}~;~Y_t = \sqrt{W_t^2(\alpha s_{t-1}^2+\sum _{i=1}^q\tilde{c}_iY_{t-i}^2)}. \end{aligned}$$

Note that $\{\tilde{c}_1,\ldots ,\tilde{c}_q\}$ represents $\{a_1,a_1b_1$ $,a_1b_1^{2},$ $\ldots ,a_1b_1^{q-1} \}$ scaled by multiplying a scalar $\frac{1-\alpha }{\sum _{j=1}^{q}a_1b_1^{j-1}}$ and the optimal combination of $\alpha , a_1,b_1$ is selected by minimizing $|KURT(W_t)-3|$ to satisfy the normalizing requirement. For the P-GE-NoVaS and P-GA-NoVaS methods, we can express $Y_{T+h}$ as a function of $\{W_{T+1},\ldots ,W_{T+h}\}$ and repeat the aforementioned procedure to obtain the optimal $L_1$ and $L_2$ predictors. For example, we can derive the expression for $Y_{T+h}$ using the P-GA-NoVaS method:

24 $$\begin{aligned} Y_{T+h} = f_{\text {P-GA}}(W_{T+1},\ldots ,W_{T+h};{\mathscr {F}}_{T})~;~\text {for any}~h\ge 1. \end{aligned}$$

#### Remark 3

(Slight computational efficiency from removing $\beta$) Note that the computation cost of NoVaS-type methods without $\beta$ term is less than that of the current ones because: recall $1/\sqrt{\beta }$ is required to be larger than or equal to three to ensure that $\{W_t\}$ has a sufficiently large range, that is, $\beta$ is required to be less than or equal to 0.111. However, the optimal combination of NoVaS coefficients may not render a suitable $\beta$. Therefore, we need to increase the time series order (*p* or *q*) and repeat the normalizing and variance-stabilizing processes until $\beta$ in the optimal combination of coefficients is appropriate. This replication process increased the computational workload.

### Connection of two parsimonious methods

In this subsection, we reveal that the P-GE-NoVaS and P-GA-NoVaS methods have the same structure. The difference between these two methods lies in the region of free parameters. To observe this phenomenon, let us consider the scaled coefficients of the P-GA-NoVaS method, except for $\alpha$.

25 $$\begin{aligned} \left\{ \frac{(1-\alpha )b_1^{i-1}}{\sum _{j=1}^{q}b_1^{j-1}}\right\} _{i=1}^{q} =\left\{ \frac{(1-\alpha )b_1^{i}}{\sum _{j=1}^{q}b_1^{j}}\right\} _{i=1}^{q}. \end{aligned}$$

Recall that the parameters of the P-GE-NoVaS method, except for $\alpha$ implied by Eq. (7), are:

26 $$\begin{aligned} \left\{ \frac{(1-\alpha )e^{-ci}}{\sum _{j=1}^pe^{-cj}} \right\} _{i=1}^{p}. \end{aligned}$$

Observing the above two equations, although we can discover that Eqs. (25) and (26) are equivalent if we set $b_1$ to be equal to $e^{-c}$, these two methods are still different because the regions $b_1$ and *c* play a role in the process of optimization. The complete region for *c* is $(0,\infty )$. However, Politis (2015) indicated that *c* cannot take a large value^8^ and the region *c* should be an interval of type (0, *m*) for some *m*. In other words, a formidable search problem for determining the optimal *c* is avoided by choosing a trimmed interval. However, $b_1$ is explicitly searched from (0, 1) which corresponds to *c* taking values from $(0,\infty )$. Similarly, by applying the P-GA-NoVaS method, the aforementioned burdensome search problem is eliminated. Moreover, we can construct a transformation based on the entire available region of the unknown parameter. Therefore, we argue that the P-GA-NoVaS method is more stable and reasonable than the P-GE-NoVaS method. Based on empirical comparisons, the P-GA-NoVaS method can achieve significantly superior prediction performance in some cases; see “Appendix 2” for more details.

### Algorithms of new methods

In this section, we provide algorithms of the two methods. For the GA-NoVaS method, the unknown parameters $\beta , a_1, b_1$ are selected from three grids of possible values to normalize $\{W_t;~t = q+1,\ldots ,T\}$. If our goal is the *h*-step-ahead prediction of $g(Y_{T+h})$ using past $\{Y_t;~t=1,\ldots ,T\}$, the algorithm of the GA-NoVaS method can be summarized in Algorithm 1.
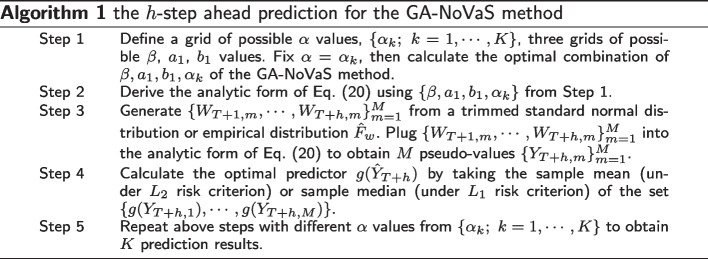


To apply the P-GA-NoVaS method, we only need to change Algorithm 1 slightly to obtain Algorithm 2.
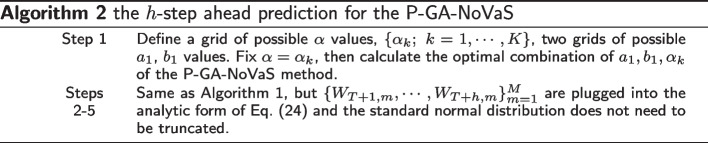


In our experimental setting, we choose regions of $\beta ,a_1,b_1$ being (0, 1) and set a 0.02 grid interval to find all parameters. In addition, for the GA-NoVaS method, we ensure that the sum of $\beta ,a_1,b_1$ is less than 1, and the coefficient of $Y_t^{2}$ is the largest.

## Simulation

In simulation studies, for controlling the dependence of prediction performance on the length of the dataset, 16 datasets (2 from each setting) are generated from 8 different GARCH(1,1)-type models separately and the size of each dataset is 250 (short data mimics 1-year of econometric data) or 500 (large data mimics 2-year of econometric data).

*Model 1* Time-varying GARCH(1,1) with Gaussian errors

$$X_t = \sigma _t\epsilon _t,~\sigma _t^2 = \omega _{0,t} + \beta _{1,t}\sigma _{t-1}^2+\alpha _{1,t}X_{t-1}^2,~\{\epsilon _t\}\sim i.i.d.~N(0,1)$$

$$g_t = t/n; \omega _{0,t}= -4sin(0.5\pi g_t)+5; \alpha _{1,t} = -1(g_t-0.3)^2 + 0.5; \beta _{1,t} = 0.2sin(0.5\pi g_t)+0.2,~n = 250~\text {or}~500$$.

*Model 2* Another time-varying GARCH(1,1) with Gaussian errors

$$X_t = \sigma _t\epsilon _t,~\sigma _t^2 = 0.00001 + \beta _{1,t}\sigma _{t-1}^2+\alpha _{1,t}X_{t-1}^2,~\{\epsilon _t\}\sim i.i.d.~N(0,1)$$

$$g_t = t/n$$; $$\alpha _{1,t} = 0.1 - 0.05g_t$$; $$\beta _{1,t} = 0.73 + 0.2g_t,~n = 250~\text {or}~500$$.

*Model 3* Standard GARCH(1,1) with Gaussian errors

$$X_t = \sigma _t\epsilon _t,~\sigma _t^2 = 0.00001 + 0.73\sigma _{t-1}^2+0.1X_{t-1}^2,~\{\epsilon _t\}\sim i.i.d.~N(0,1)$$.

*Model 4* Standard GARCH(1,1) with Gaussian errors

$$X_t = \sigma _t\epsilon _t,~\sigma _t^2 = 0.00001 + 0.8895\sigma _{t-1}^2+0.1X_{t-1}^2,~\{\epsilon _t\}\sim i.i.d.~N(0,1)$$.

*Model 5* Standard GARCH(1,1) with Student-*t* errors

$$X_t = \sigma _t\epsilon _t,$$ $$~\sigma _t^2 = 0.00001 + 0.73\sigma _{t-1}^2+0.1X_{t-1}^2,$$

$$~\{\epsilon _t\}\sim i.i.d.~t$$ $$\text {distribution with five degrees of freedom}$$.

*Model 6* Exponential GARCH(1,1) with Gaussian errors

$$X_t = \sigma _t\epsilon _t,~\log (\sigma _t^2) = 0.00001 + 0.8895\log (\sigma ^2_{t-1})+0.1\epsilon _{t-1}+0.3(|\epsilon _{t-1}|-E|\epsilon _{t-1}|),$$

$$~\{\epsilon _t\}\sim i.i.d.~N(0,1)$$.

*Model 7* GJR-GARCH(1,1) with Gaussian errors

$$X_t = \sigma _t\epsilon _t,~\sigma _t^2 = 0.00001 + 0.5\sigma ^2_{t-1}+0.5X_{t-1}^2-0.5I_{t-1}X_{t-1}^2,~\{\epsilon _t\}\sim i.i.d.~N(0,1)$$

$$I_{t} = 1~\text {if}~ X_t \le 0, and I_{t} = 0~ \text {otherwise}$$.

*Model 8* Another GJR-GARCH(1,1) with Gaussian errors

$$X_t = \sigma _t\epsilon _t,~\sigma _t^2 = 0.00001 + 0.73\sigma ^2_{t-1}+0.1X_{t-1}^2+0.3I_{t-1}X_{t-1}^2,~\{\epsilon _t\}\sim i.i.d.~N(0,1)$$

$$I_{t} = 1~\text {if}~ X_t \le 0, and I_{t} = 0~ \text {otherwise}$$.

*Model description* Models 1 and 2 present a time-varying GARCH model where coefficients $a_0, a_1, b_1$ change over time slowly. They differ significantly in the intercept term of $\sigma _t^2$ as we intentionally kept it low in the second setting. Models 3 and 4 are from a standard GARCH, where, in Model 4, we wanted to explore a scenario in which $\alpha _1+\beta _1$ is very close to 1 and thus mimics what would happen for the iGARCH situation. Model 5 allows the error distribution to originate from a student-*t* distribution instead of a Gaussian distribution. For fair competition with the existing GE-NoVaS method, we chose Models 2 to 5, similar to the simulation settings of Chen and Politis (2019). Models 6, 7, and 8 present the different types of GARCH models. These settings allow us to check the robustness of our method against model misspecification. In the real world, it is difficult to convincingly tell if the data obey one particular type of GARCH model; hence, we shall pursue this exercise to see if our methods are satisfactory, regardless of the underlying distribution and the GARCH-type model. This approach to test the performance of a method under model misspecification is standard; see Olubusoye et al. (2016) and Bellini and Bottolo (2008) for more examples.

*Window size* Using these datasets, we perform 1-step, 5-steps, and 30-steps ahead time-aggregated POOS predictions. To measure the prediction performance of different methods on larger datasets (i.e., data size of 500), we use 250 data as a window to perform predictions and roll this window through the entire dataset. To evaluate the performance of different methods on smaller datasets (i.e., data size of 250), we use 100 data as a window.

*Different variants of methods* Note that we can perform GE-NoVaS-type and GA-NoVaS-type methods to predict $Y_{T+h}$ by generating $\{W_{T+1,m},\ldots , W_{T+h,m}\}_{m=1}^{M}$ from a standard normal distribution or the empirical distribution of $\{W_t\}$ series, then we can calculate the optimal predictor based on $L_1$ or $L_2$ risk criterion. This means that each NoVaS-type method has four variants.

When performing POOS forecasting, we do not know which $\alpha$ is optimal. Therefore, we perform every NoVaS variant using $\alpha$ from eight potential values $\{0.1, 0.2, \ldots ,0.8\}$ and then select the optimal result. To simplify the presentation, we further select the final prediction from the optimal results of the four variants of the NoVaS method and use this result to be the best prediction to which each NoVaS method can reach. This procedure allows us to take a computationally intensive approach to compare the potentially best performances of different methods.

### Simulation results

In this subsection, we compare the performances of our new methods (GA-NoVaS and P-GA-NoVaS) with GARCH-direct and existing GE-NoVaS methods on forecasting 250 and 500 simulated data. Based on the time-aggregated prediction metric Eq. (16), the results are tabulated in Table 1.^9^

#### Simulation results of Models 1 to 5

From Table 1, we conclusively find that NoVaS-type methods outperform the GARCH-direct method. Especially when using the 500 Model 1 data to perform 30-steps ahead of the aggregated prediction, the performance of the GARCH-direct method is poor. NoVaS-type methods are almost 30 times better than the GARCH-direct method indicating that the standard prediction method may be affected by the error accumulation problem when long-term predictions are required. However, model-free methods can overcome this problem.

In addition to the overall advantage of NoVaS-type methods over the GARCH-direct method, we find that the GA-NoVaS method is generally better than the GE-NoVaS method in predicting both short and large data. This conclusion is two-fold: (1) GA-NoVaS consumes less time than the GE-NoVaS method; (2) because we want to compare the forecasting ability of the GE-NoVaS and GA-NoVaS methods, we use $*$ symbol to represent cases where the GA-NoVaS method works at least 10$\%$ better than the GE-NoVaS method, or inversely, the GE-NoVaS method is 10$\%$ better. We find no case to support that the GE-NoVaS works better than the GA-NoVaS with at least 10$\%$ improvement. On the other hand, the GA-NoVaS method exhibits a significant improvement when long-term predictions are required. Moreover, the P-GA-NoVaS dominates the other two NoVaS-type methods.

#### Models 6 to 8: different GARCH specifications

Since the main crux of Model-free methods is how such non-parametric methods are robust to different underlying data-generation processes. The GA-NoVaS method is based on the GARCH model, so it is interesting to explore whether these methods can sustain a different type of true underlying data generation process. The simulation results for Models 6–8 are tabulated in Table 1.

In general, NoVaS-type methods still outperform the GARCH-direct method for these cases. The GA-NoVaS method is better than the GE-NoVaS method in terms of long-term forecasting. In addition, the GA-NoVaS method can also bring about significant improvement for short-size data, such as the 30-steps ahead aggregated prediction of 250 Model 6 simulated data. Improving prediction with short data is always a significant challenge; thus, it is valuable to discover whether the GA-NoVaS method gives superior performance in this scenario. Unsurprisingly, the P-GA-NoVaS method performed well.

### Simulation summary

Simulation data analysis shows that NoVaS-type methods can sustain great performance against short data and model misspecification. Overall, our new method outperforms the GE-NoVaS method with notable improvements in some cases where long-term predictions are desired, such as the 500-size simulation of Model 8. Table 1, clearly shows that the GARCH-direct method is unsuitable for this case. To further compare the different methods in an absolute sense for this case, we plot the predictions of different methods and actual values in the same figure. Based on these plots, it is clear that the GARCH method is unstable and far from the true curve for long-term aggregated predictions. However, NoVaS-type methods work well and fit the trend of the true curve in an absolute sense. The corresponding plots are shown in “Appendix 3”. Furthermore, the NoVaS-type methods outperform the GARCH method for Models 3 and 4, even if the underlying model is also GARCH(1,1). Moreover, we find that NoVaS-type methods are competitive when applying the estimated Exponential GARCH(1,1) and GJR-GARCH(1,1) models to predict Models 6 and 8, respectively. These results further support the claim that NoVaS-type methods are robust against model misspecification. The efficiency of the model-free prediction concept is demonstrated. The corresponding analyses are provided in “Appendix 3”.

**Table 1:** Comparison results of using 500 and 250 simulated data

500 size	GE	GA	P-GA	GARCH	250 size	GE	GA	P-GA	GARCH
M1-1step	0.98241	0.97574	**0.91931**	1.00000	M1-1step	0.91538	0.91120	**0.83034**	1.00000
M1-5steps	0.75332	0.75716	**0.70509**	1.00000	M1-5steps	0.49169	0.48479	**0.43247**	1.00000
M1-30steps	0.16629	0.16108	**0.15950**	1.00000	M1-30steps	0.25009	0.24752	**0.23035**	1.00000
M2-1step	0.97948	**0.97391**	0.98569	1.00000	M2-1step	0.91369	0.91574	**0.87614**	1.00000
M2-5steps	0.91726	**0.88645**	0.91872	1.00000	M2-5steps	0.61001	0.61094	**0.51712**	1.00000
M2-30steps	0.78096	**0.72696**	0.80259	1.00000	M2-30steps	0.7725	**0.74083**	0.75251	1.00000
M3-1step	1.00334	0.99333	**0.98694**	1.00000	M3-1step	0.97796	0.96632	**0.93693**	1.00000
M3-5steps	0.98281	0.98312	**0.96297**	1.00000	M3-5steps	0.98127	**0.97897**	0.99977	1.00000
M3-30steps	0.74129	**0.72216**	0.72611	1.00000	M3-30steps	1.38353	0.89001*	0.99818	1.00000
M4-1step	1.00390	**0.98736**	1.04656	1.00000	M4-1step	0.99183	0.95698	**0.92811**	1.00000
M4-5steps	0.98563	**0.94474**	1.16346	1.00000	M4-5steps	0.77088	0.72882	**0.67894**	1.00000
M4-30steps	**0.75828**	0.79120	0.77923	1.00000	M4-30steps	0.79672	0.60950*	0.81115	1.00000
M5-1step	0.71259	**0.70934**	0.72124	1.00000	M5-1step	0.83631	0.84134	0. **79075**	1.00000
M5-5steps	0.31050	**0.30918**	0.31147	1.00000	M5-5steps	0.38296	0.38034	**0.35155**	1.00000
M5-30steps	0.01088	0.01087	**0.01069**	1.00000	M5-30steps	0.00199	0.00201	**0.00194**	1.00000
M6-1step	1.02897	1.02439	**0.97537**	1.00000	M6-1step	0.95939	0.96499	**0.93863**	1.00000
M6-5steps	1.02441	1.02850	**0.93029**	1.00000	M6-5steps	0.93594	0.97101	**0.85851**	1.00000
M6-30steps	1.03845	1.03545	**0.89686**	1.00000	M6-30steps	0.84401	0.67272*	0.70420	1.00000
M7-1step	0.98459	0.98179	**0.96345**	1.00000	M7-1step	0.84813	0.83628	**0.83216**	1.00000
M7-5steps	0.98910	0.99239	**0.96265**	1.00000	M7-5steps	0.50849	0.50126	**0.48020**	1.00000
M7-30steps	0.74522	0.73544	**0.64106**	1.00000	M7-30steps	0.06832	0.06817	**0.06507**	1.00000
M8-1step	**0.79801**	0.80723	0.81895	1.00000	M8-1step	**0.79561**	0.79994	0.83340	1.00000
M8-5steps	0.34915	**0.34223**	0.35644	1.00000	M8-5steps	0.48028	0.47244	**0.45665**	1.00000
M8-30steps	0.00106	0.00087*	0.00094	1.00000	M8-30steps	0.00977	**0.00942**	0.00983	1.00000

Column names “GA” and “GE” represent GE-NoVaS and GA-NoVaS methods, respectively; “GARCH” represents the GARCH-direct method; and “P-GA” represents the P-GA-NoVaS method. The benchmark is the GARCH-direct method, so numerical values in the table corresponding to the GARCH-direct method are 1. Other numerical values are relative performance compared to the GARCH-direct method. “$Mi\text {-}j$”steps denotes using data generated from the Model *i* to do *j* steps ahead time-aggregated predictions. The bold value means that the corresponding method is the optimal choice for this data case. Cell with $*$ means that the GA-NoVaS method is at least 10$\%$ better than the GE-NoVaS method, or inversely, the GE-NoVaS method is at least 10$\%$ better

## Real-world data analysis

This section is devoted to exploring, in the context of real datasets forecasting, whether NoVaS-type methods can provide good long-term time-aggregated forecasting and how our new methods compare to the existing Model-free method.

To conduct extensive analyses and subsequently obtain a convincing conclusion, we use three types of data—stock, index, and currency—to perform predictions. Moreover, as in the simulation studies, we apply this exercise to two different data lengths. To build large datasets (2-year period data), we take more recent datasets from January 2018 to December 2019 and previous data from approximately 20 years ago, separately. The dynamics of these econometric datasets have changed significantly over the past 20 years; therefore, we wanted to explore whether our methods are suitable for both old and new data. Subsequently, we challenge our methods using short (1-year) real-life data. We also perform forecasting using volatile data, that is, data from November 2019 to October 2020. Note that economies across the world went through a recession due to the COVID-19 pandemic and then slowly recovered during this period; typically, these types of situations introduce systematic perturbation in the dynamics of econometric datasets. We aimed to determine whether our methods could sustain such perturbations or abrupt changes.

### 2-year data

For mimicking the 2-year period data, we adopt several stock datasets—AAPL, BAC, MSFT and MCD—with 500 data size to perform forecasting. In summary, we compare the performances of different methods on 1-step, 5-steps, and 30-steps ahead POOS time-aggregated predictions. All results obtained through a procedure similar to that in “Simulation” section are shown in Table 2. The NoVaS-type methods still outperform the GARCH-direct method. Additionally, our new method is more robust than the GE-NoVaS method; see the 30-steps ahead prediction of the previous 2-year BAC and MSFT cases. We can also see that the P-GA-NoVaS method is more robust than the other two NoVaS methods. The $\beta$-removing idea proposed by Wu and Karmakar (2021) was substantiated again.

Because the main objective of this study is to offer a new type of NoVaS method that performs better than the GE-NoVaS method in dealing with short and volatile data, we provide more extensive data analyses to support our new methods in the sections ahead.

**Table 2:** Comparison results of using 2-year data

Previous 2-year	GE	GA	P-GA	GARCH	More recent 2-year	GE	GA	P-GA	GARCH
AAPL-1step	0.99795	0.99236	**0.97836**	1.00000	AAPL-1step	0.80150	**0.79899**	0.79915	1.00000
AAPL-5steps	1.04919	1.04800	**0.96999**	1.00000	AAPL-5steps	0.41405	0.42338	**0.40427**	1.00000
AAPL-30steps	1.12563	1.21986	**0.96174**	1.00000	AAPL-30steps	**0.13207**	0.14046	0.14543	1.00000
BAC-1step	**0.99889**	1.00396	1.02780	1.00000	BAC-1step	0.98393	0.99164	**0.96542**	1.00000
BAC-5steps	1.04424	1.02185	**0.99399**	1.00000	BAC-5steps	0.98885	1.01480	**0.91857**	1.00000
BAC-30steps	1.32452	1.13887*	1.00363	**1.00000**	BAC-30steps	1.14111	1.03657	**0.88596**	1.00000
MSFT-1step	0.98785	0.98598	**0.96185**	1.00000	MSFT-1step	0.98405	0.98630	**0.96374**	1.00000
MSFT-5steps	1.00236	1.00096	**0.95271**	1.00000	MSFT-5steps	0.65027	0.67005	**0.64278**	1.00000
MSFT-30steps	1.25272	1.09881*	**0.88515**	1.00000	MSFT-30steps	**0.19767**	0.20060	0.21473	1.00000
MCD-1step	1.01845	1.00789	**0.99005**	1.00000	MCD-1step	0.99631	0.99539	**0.98035**	1.00000
MCD-5steps	1.11249	1.07748	**0.97777**	1.00000	MCD-5steps	0.95403	0.95327	**0.91317**	1.00000
MCD-30steps	1.76385	1.69757	**0.99418**	1.00000	MCD-30steps	0.75730	0.75361	**0.74557**	1.00000

Column names “GA” and “GE” represent GE-NoVaS and GA-NoVaS methods, respectively; “GARCH” means the GARCH-direct method; and “P-GA” means the P-GA-NoVaS method. The benchmark is the GARCH-direct method, so numerical values in the table corresponding to the GARCH-direct method are 1. Other numerical values are relative performance compared to the GARCH-direct method. The bold value indicates that the corresponding method is the optimal choice for this data case. A cell with $*$ indicates that the GA-NoVaS method is at least 10$\%$ better than the GE-NoVaS method, or inversely, the GE-NoVaS method is at least 10$\%$ better

### 2018 and 2019 1-year data

For challenging our new methods in contrast to other methods for small real-life datasets, we separate every new 2-year period data in “2-year data” section into two 1-year period datasets, that is, separate four new stock datasets to eight samples. We believe that evaluating the prediction performance using shorter data is a more important problem, and thus, we wanted to make our analysis very comprehensive. Therefore, for this exercise, we add seven index datasets: NASDAQ, NYSE, Small Cap, Dow Jones, S &P 500, BSE and BIST; and two stock datasets: Tesla and Bitcoin into our analysis.

From Table 3, which presents the prediction results of different methods on the 2018 and 2019 stock data, we still observe that NoVaS-type methods outperform the GARCH-direct method for almost all cases. Among the different NoVaS methods, it is obvious that our new methods are superior to the existing GE-NoVaS method. After applying the $\beta$-removing concept, the P-GA-NoVaS method significantly outperforms the other methods in almost all cases.

From Table 4, which presents the prediction results of different methods on the 2018 and 2019 index data, we obtain the same conclusion as before. NoVaS-type methods are far superior to the GARCH-direct and our new NoVaS methods outperform the existing GE-NoVaS method. Interestingly, the GE-NoVaS method is beaten by the GARCH-direct method in some cases, such as the 2019-NASDAQ, Smallcap, and BIST. However, the new methods still exhibit stable performance.

**Table 3:** Comparison results of using 2018 and 2019 stock data

2018	GE	GA	P-GA	GARCH	2019	GE	GA	P-GA	GARCH
MCD-1step	0.98514	0.97887	**0.94412**	1.00000	MCD-1step	0.95959	0.96348	**0.94559**	1.00000
MCD-5steps	1.02720	1.02519	**0.88151**	1.00000	MCD-5steps	1.00723	1.01169	**0.90602**	1.00000
MCD-30steps	0.62614	0.63992	**0.61153**	1.00000	MCD-30steps	1.05239	0.95714	**0.77976**	1.00000
AAPL-1step	0.92014	0.92317	**0.89283**	1.00000	AAPL-1step	0.84533	**0.81326**	0.81872	1.00000
AAPL-5steps	0.84798	0.73461*	**0.71233**	1.00000	AAPL-5steps	0.85401	0.79254	**0.68792**	1.00000
AAPL-30steps	0.38612	**0.36324**	0.37081	1.00000	AAPL-30steps	0.99043	0.99286	**0.72892**	1.00000
BAC-1step	0.94952	0.93842	0**.92619**	1.00000	BAC-1step	1.04272	1.04722	**0.98605**	1.00000
BAC-5steps	0.83395	0.79158	**0.72512**	1.00000	BAC-5steps	1.22761	1.20195	**0.95436**	1.00000
BAC-30steps	1.34367	0.90675*	**0.87630**	1.00000	BAC-30steps	1.45020	1.41788	1.03482	**1.00000**
MSFT-1step	0.91705	**0.90936**	0.95921	1.00000	MSFT-1step	1.03308	1.00101	**0.95347**	1.00000
MSFT-5steps	0.74553	0.74267	**0.74237**	1.00000	MSFT-5steps	1.22340	1.18205	**0.95417**	1.00000
MSFT-30steps	0.66990	0.64770	**0.64717**	1.00000	MSFT-30steps	1.23020	1.21337	**0.98476**	1.00000
Tesla-1step	1.00181	0.96074	**0.86238**	1.00000	Tesla-1step	1.00428	1.01934	**0.98955**	1.00000
Tesla-5steps	1.20383	1.13335	1.01560	**1.00000**	Tesla-5steps	1.06610	1.07506	**0.96107**	1.00000
Tesla-30steps	1.97328	1.84871	1.25005	**1.00000**	Tesla-30steps	2.00623	1.71782*	**0.84366**	1.00000
Bitcoin-1step	0.99636	1.01731	**0.97734**	1.00000	Bitcoin-1step	0.89929	0.88914	**0.87256**	1.00000
Bitcoin-5steps	1.02021	1.11880	**0.93826**	1.00000	Bitcoin-5steps	0.62312	0.63075	**0.56789**	1.00000
Bitcoin-30steps	**0.86649**	0.95506	0.91364	1.00000	Bitcoin-30steps	0.00733	0.00749	**0.00631**	1.00000

Column names “GA” and “GE” represent GE-NoVaS and GA-NoVaS methods, respectively; “GARCH” means the GARCH-direct method; and “P-GA” means the P-GA-NoVaS method. The benchmark is the GARCH-direct method, so numerical values in the table corresponding to the GARCH-direct method are 1. Other numerical values are relative performance compared to the GARCH-direct method. The bold value indicates that the corresponding method is the optimal choice for this data case. Cell with $*$ indicate that the GA-NoVaS method is at least 10$\%$ better than the GE-NoVaS method, or inversely, the GE-NoVaS method is at least 10$\%$ better

**Table 4:** Comparison results of using 2018 and 2019 index data

2018	GE	GA	P-GA	GARCH	2019	GE	GA	P-GA	GARCH
NASDAQ-1step	**0.91309**	0.92303	0.92421	1.00000	NASDAQ-1step	0.99960	0.98950	**0.93843**	1.00000
NASDAQ-5steps	**0.76419**	0.79718	0.78823	1.00000	NASDAQ-5steps	1.15282	1.09176	**0.84051**	1.00000
NASDAQ-30steps	0.66520	**0.65489**	0.67389	1.00000	NASDAQ-30steps	0.68994	0.69846	**0.59218**	1.00000
NYSE-1step	0.93509	**0.93401**	0.96619	1.00000	NYSE-1step	0.92486	**0.91118**	0.92193	1.00000
NYSE-5steps	0.83725	0.79330	**0.75822**	1.00000	NYSE-5steps	0.86249	0.82114	**0.71038**	1.00000
NYSE-30steps	0.75053	0.61443*	0.61830	1.00000	NYSE-30steps	0.22122	0.22173	**0.18116**	1.00000
Smallcap-1step	**0.90546**	0.91346	0.91101	1.00000	Smallcap-1step	1.02041	1.00626	**0.98482**	1.00000
Smallcap-5steps	**0.72627**	0.73955	0.73223	1.00000	Smallcap-5steps	1.15868	1.08929	**0.85490**	1.00000
Samllcap-30steps	0.50005	0.46482	**0.46312**	1.00000	Samllcap-30steps	1.30467	1.28949	**0.90360**	1.00000
Djones-1step	0.90932	**0.90707**	0.91192	1.00000	Djones-1step	0.96752	**0.96433**	0.96977	1.00000
Djones-5steps	0.82480	0.79965	**0.76226**	1.00000	Djones-5steps	0.98725	0.93315	**0.91238**	1.00000
Djones-30steps	0.72547	0.53021*	0.56854	1.00000	Djones-30steps	0.86333	0.85006	**0.81803**	1.00000
S &P500-1step	0.91860	0.91256	**0.88405**	1.00000	S &P500-1step	0.96978	0.96526	**0.93162**	1.00000
S &P500-5steps	0.85108	0.77305	**0.75646**	1.00000	S &P500-5steps	0.96704	0.94028	**0.77434**	1.00000
S &P500-30steps	0.88917	0.68156*	0.72104	1.00000	S &P500-30steps	0.34389	0.34537	**0.30127**	1.00000
BSE-1step	0.99942	0.88322*	0.92568	1.00000	BSE-1step	0.70667	0.70194	**0.66667**	1.00000
BSE-5steps	0.92061	0.78484*	0.84408	1.00000	BSE-5steps	0.25675	0.25897	**0.23603**	1.00000
BSE-30steps	0.52431	0.41010*	0.44092	1.00000	BSE-30steps	0.03764	0.03951	**0.02888**	1.00000
BIST-1step	0.93221	**0.92215**	0.94138	1.00000	BIST-1step	**0.96807**	0.97209	0.98234	1.00000
BIST-5steps	0.82149	**0.79664**	0.81417	1.00000	BIST-5steps	0.98944	1.03903	**0.85370**	1.00000
BIST-30steps	1.34581	1.42233	1.09900	**1.00000**	BIST-30steps	2.21996	2.10562	**0.85743**	1.00000

Column names “GA” and “GE” represent GE-NoVaS and GA-NoVaS methods, respectively; “GARCH” means the GARCH-direct method; and “P-GA” means the P-GA-NoVaS method. As the benchmark is the GARCH-direct method, numerical values in the table corresponding to the GARCH-direct method are 1. Other numerical values are relative performance compared to the GARCH-direct method. The bold value indicates that the corresponding method is the optimal choice for this data case. A cell with $*$ indicates that the GA-NoVaS method is at least 10$\%$ better than the GE-NoVaS method, or inversely, the GE-NoVaS method is at least 10$\%$ better

### Volatile 1-year data

**Fig. 1:**
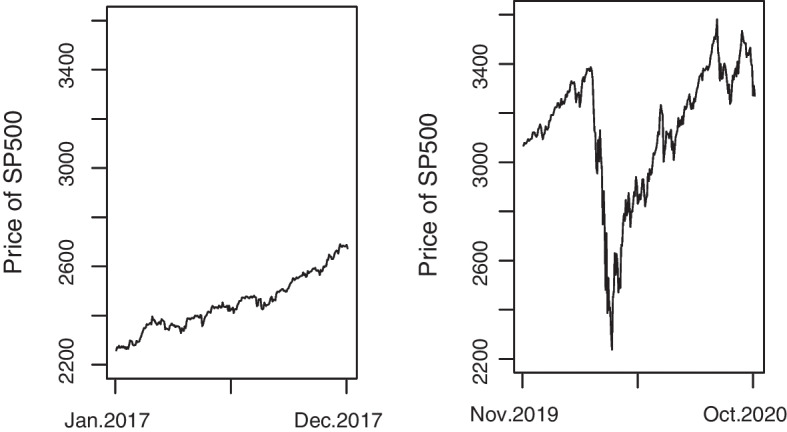
The left subfigure depicts the price of S &P500 from January 2017 to December 2017 which presents a slow growth; The right subfigure depicts the price of S &P500 from November 2019 to October 2020

In this subsection, we perform POOS forecasting using volatile 1-year data (i.e., data from November 2019 to October 2020). We tactically choose this period data to challenge our new methods for checking whether it can self-adapt to the structural incoherence between pre- and post-pandemic, and compare our new methods with the existing GE-NoVaS method. To observe the effects of the pandemic, we take the price of the S &P500 index as an example. From Fig. 1, it is apparent that the price grew slowly during the normal period from January 2017 to December 2017. However, during the period from November 2019 to October 2020, prices fluctuated severely due to the pandemic.

#### Stock data

The POOS forecasting results of volatile 1-year stock datasets are presented in Table 5. NoVaS-type methods dominate the GARCH-direct method. The performance of the GARCH-direct method is poor, especially for the Bitcoin case. Apart from this overall advantage of NoVaS-type methods, there is no doubt that the GA-NoVaS method exhibits greater prediction results than the GE-NoVaS method because it occupies 13 out of 27 optimal choices and represents at least a 10$\%$ improvement for five cases. The P-GA-NoVaS method also shows better results than those of the GE-NoVaS method.

**Table 5:** Comparison results of using volatile 1-year stock data

	GE-NoVaS	GA-NoVaS	P-GA-NoVaS	GARCH-direct
NKE-1step	0.63568	**0.63209**	0.65594	1.00000
NKE-5steps	0.20171	**0.19089**	0.22226	1.00000
NKE-30steps	0.00411	0.00278*	0.00340	1.00000
AMZN-1step	0.97099	0.96719	**0.90487**	1.00000
AMZN-5steps	0.88705	0.88274	**0.72850**	1.00000
AMZN-30steps	0.58124	0.62863	**0.53310**	1.00000
IBM-1step	0.80222	0.79823	**0.79509**	1.00000
IBM-5steps	0.38933	**0.37346**	0.38413	1.00000
IBM-30steps	0.01143	0.00996*	**0.00879**	1.00000
MSFT-1step	0.80133	**0.79528**	0.81582	1.00000
MSFT-5steps	0.35567	**0.33419**	0.38022	1.00000
MSFT-30steps	0.01342	0.01031*	**0.00784**	1.00000
SBUX-1step	0.68206	0.67067	**0.66743**	1.00000
SBUX-5steps	0.24255	**0.23072**	0.26856	1.00000
SBUX-30steps	0.00499	0.00337*	**0.00236**	1.00000
KO-1step	0.77906	**0.75389**	0.77035	1.00000
KO-5steps	0.34941	**0.32459**	0.33405	1.00000
KO-30steps	0.01820	0.01848	**0.01582**	1.00000
MCD-1step	0.51755	**0.51351**	0.56414	1.00000
MCD-5steps	0.10725	**0.09714**	0.17439	1.00000
MCD-30steps	3.32E−05	2.97E−05*	**7.62E**−**06**	1.00000
Tesla-1step	0.90712	0.90250	**0.88782**	1.00000
Tesla-5steps	0.68450	0.67935	**0.66937**	1.00000
Tesla-30steps	**0.21643**	0.21718	0.22395	1.00000
Bitcoin-1step	0.36323	**0.36260**	0.36326	1.00000
Bitcoin-5steps	**0.01319**	0.01321	0.01322	1.00000
Bitcoin-30steps	7.75E−17	**7.65E**−**17**	7.75E−17	1.00000

As the benchmark is the GARCH-direct method, numerical values in the table corresponding to the GARCH-direct method are 1. Other numerical values are relative performance compared to the GARCH-direct method. The bold value means that the corresponding method is the optimal choice for this data case. Cell with $*$ means that the GA-NoVaS method is at least 10$\%$ better than the GE-NoVaS method, or inversely, the GE-NoVaS method is at least 10$\%$ better

#### Currency data

The POOS forecasting results of selected most recent 1-year currency datasets are presented in Table 6. The meaning of bold values and values with asterisk marks is the same as the definition in Table 5. Note that Fryzlewicz et al. (2008) showed that the ARCH framework appears to be a superior methodology for dealing with currency exchange data. Therefore, we should not anticipate that GA-NoVaS-type methods can attain significant improvements for this data case. However, the GA-NoVaS method still results in approximately 26$\%$ and 37$\%$ improvement for 30-steps ahead aggregated predictions of CADJPY and CNYJPY, respectively. Besides, the P-GA-NoVaS method also remains a great performance.

**Table 6:** Comparison results of using volatile 1-year currency data

	GE-NoVaS	GA-NoVaS	P-GA-NoVaS	GARCH-direct
CADJPY-1step	0.46940	**0.46382**	0.48367	1.00000
CADJPY-5steps	0.11678	**0.11620**	0.14376	1.00000
CADJPY-30steps	0.00584	0.00430*	0.00482	1.00000
EURJPY-1step	0.95093	**0.94682**	0.95133	1.00000
EURJPY-5steps	0.76182	0.77091	**0.75636**	1.00000
EURJPY-30steps	**0.16202**	0.17956	0.18189	1.00000
USDCNY-1step	0.98905	0.97861	**0.95757**	1.00000
USDCNY-5steps	0.93182	0.92614	**0.83523**	1.00000
USDCNY-30steps	0.57171	**0.57100**	0.60131	1.00000
GBPJPY-1step	0.86971	**0.86474**	0.87160	1.00000
GBPJPY-5steps	0.49749	0.49612	**0.48842**	1.00000
GBPJPY-30steps	0.17058	**0.16987**	0.17262	1.00000
USDINR-1step	0.97289	0.96829	**0.93140**	1.00000
USDINR-5steps	0.80866	0.78008	**0.75693**	1.00000
USDINR-30steps	**0.09725**	0.09889	0.11380	1.00000
CNYJPY-1step	0.77812	0.77983	**0.74586**	1.00000
CNYJPY-5steps	0.38875	0.38407	**0.34839**	1.00000
CNYJPY-30steps	0.08398	0.05240*	0.05444	1.00000

#### Index data

The POOS forecasting results of the most recent 1-year index datasets are presented in Table 7. The meaning of bold values and values with asterisk marks is the same as the definition in Table 5. Consistent with the conclusions corresponding to the previous two classes of data, NoVaS-type methods still exhibit obviously better performance than the GARCH-direct method. In addition to this advantage of NoVaS methods, new methods still govern the existing GE-NoVaS method. In addition to these expected results, we find that the GE-NoVaS method is 14$\%$ worse than the GARCH-direct method for 1-step USDX future case. However, GA-NoVaS-type methods still perform well. This phenomenon also appears in “Simulation results of Models 1 to 5”, “Models 6 to 8: Different GARCH specifications”, “Simulation summary”, “2-year data” and “2018 and 2019 1-year data” sections. Beyond this, there are 12 cases in which the GA-NoVaS method renders more than a 10$\%$ improvement compared to the GE-NoVaS method.

**Table 7:** Comparison results of using volatile 1-year index data

	GE-NoVaS	GA-NoVaS	P-GA-NoVaS	GARCH-direct
S &P500-1step	0.97294	0.95881	**0.92854**	1.00000
S &P500-5steps	0.96590	0.94457	**0.77060**	1.00000
S &P500-30steps	0.34357	0.34561	**0.30115**	1.00000
NASDAQ-1step	0.71380	**0.70589**	0.77753	1.00000
NASDAQ-5steps	0.29332	**0.27007**	0.36428	1.00000
NASDAQ-30steps	0.01223	0.00618*	0.00696	1.00000
NYSE-1step	0.55741	0.55548	**0.54598**	1.00000
NYSE-5steps	0.08994	0.07666*	0.07798	1.00000
NYSE-30steps	1.36E−05	9.06E−06*	**6.57E**−**06**	1.00000
Smallcap-1step	0.58170	**0.57392**	0.57773	1.00000
Smallcap-5steps	0.10270	0.10135	**0.09628**	1.00000
Smallcap-30steps	7.00E−05	4.33E−05*	**3.65E**−**05**	1.00000
BSE-1step	0.39493	**0.37991**	0.39851	1.00000
BSE-5steps	0.03320	0.02829*	0.04170	1.00000
BSE-30steps	2.45E−05	2.19E−05*	**1.73E**−**05**	1.00000
DAX-1step	**0.65372**	0.65663	0.66097	1.00000
DAX-5steps	0.10997	**0.10828**	0.11085	1.00000
DAX-30steps	4.97E−05	**4.87E**−**05**	7.81E−05	1.00000
USDX future-1step	1.14621	1.00926*	1.03693	**1.00000**
USDX future-5steps	0.61075	0.53834*	**0.51997**	1.00000
USDX future-30steps	0.10723	**0.09911**	0.10063	1.00000
Bovespa-1step	0.60031	**0.57316**	0.60656	1.00000
Bovespa-5steps	0.08603	0.06201*	0.09395	1.00000
Bovespa-30steps	6.87E−06	**2.82E**−06*	3.19E−06	1.00000
Djones-1step	0.56357	0.55020	**0.54422**	1.00000
Djones-5steps	0.09810	0.08239*	0.08698	1.00000
Djones-30steps	4.32E−05	**2.22E**−05*	2.65E−05	1.00000
BIST-1step	0.94794	0.95313	**0.92418**	1.00000
BIST-5steps	**0.48460**	0.49098	0.49279	1.00000
BIST-30steps	**0.05478**	0.05980	0.05671	1.00000

### Summary of real-world data analysis

After extensive real-world data analysis, we can conclude that NoVaS-type methods generally perform better than the GARCH-direct method. Sometimes, the long-term prediction of the GARCH-direct method is impaired because of accumulated errors. Applying NoVaS-type methods helps avoid this issue. In addition to this encouraging result, the two new NoVaS methods proposed in this study perform better than the existing GE-NoVaS method, especially for analyzing short and volatile data. We present some plots to compare various methods in “Appendix 4”, as we did in “Simulation summary” section. In addition, the satisfactory performance of NoVaS-type methods in predicting Bitcoin data may also open up the application of NoVaS-type methods to forecasting cryptocurrency data.

## Comparison of predictive accuracy: statistical tests

In this section, we determine whether the victory of our new methods is statistically significant. We note that Wu and Karmakar (2021) applied CW tests to show that removing the-$\beta$ idea is appropriate for refining the GE-NoVaS method. Likewise, we are curious whether this refinement is reasonable for deriving the P-GA-NoVaS method from the GA-NoVaS method. In this study, we focus on the CW test built by Clark and West (2007)^10^ which applied an adjusted Mean Squared Prediction Error (MSPE) statistic to test if the parsimonious null model and larger model have equal predictive accuracy; see Dangl and Halling (2012), Kong et al. (2011) and Dai and Chang (2021) for examples of applying this CW test. In addition, we also take the Model Confidence Set (MCS) proposed by Hansen et al. (2011) to eliminate inferior models.

### CW-test

Note that the P-GA-NoVaS method is parsimonious compared to the GA-NoVaS method. The reason for removing the $\beta$ term is described in “Motivations of building a new NoVaS transformation” section. Here, we want to deploy the CW test to ensure that the $\beta$-removing idea is not only empirically adoptable, but also statistically reasonable. We use several results from “Real-world data analysis” section  to run the CW tests. However, it is difficult to apply the CW test to compare 5-steps and 30-steps aggregated predictions. In other words, the CW test results for the aggregated predictions are ambiguous. It is difficult to explain the significance of a significantly small *p* value. Does this mean that the method outperforms the opposite for all single-step horizons? Alternatively, does this mean that the method achieves better performance in some specific future steps? Therefore, we consider the 1-step ahead prediction horizon, and the CW test results are tabulated in Table 8.

From Table 8, under a one-sided 5$\%$ significance level, there is only one case out of the 28 cases that reject the null hypothesis. This result implies that GA-NoVaS and P-GA-NoVaS methods are statistically equivalent. However, the P-GA-NoVaS method is more computationally efficient than the GA-NoVaS method because it uses a more concise format. More importantly, it provides a better empirical prediction performance. Thus, the reasonability of removing $\beta$ term is demonstrated again, and we favor the P-GA-NoVaS method in practice.

**Table 8:** CW-tests on 1-step ahead predictions of GA-NoVaS and P-GA-NoVaS methods

	*P* value	GA-NoVaS performance	P-GA-NoVaS performance
2018-AAPL-1step	0.99	0.92	0.89
2019-AAPL-1step	0.08	0.81	0.82
2018-BAC-1step	0.63	0.94	0.93
2019-BAC-1step	0.49	1.05	0.99
2018-TSLA-1step	0.27	0.92	0.86
2019-TSLA-1step	0.22	1.02	0.99
2018-MCD-1step	0.57	0.98	0.94
2019-MCD-1step	0.19	0.96	0.95
2018-MSFT-1step	0.17	0.91	0.96
2019-MSFT-1step	0.47	1.00	0.95
2018-Djones-1step	0.64	0.91	0.91
2019-Djones-1step	0.27	0.96	0.97
2018-NASDAQ-1step	0.51	0.92	0.92
2019-NASDAQ-1step	0.48	0.99	0.94
2018-NYSE-1step	0.31	0.93	0.97
2019-NYSE-1step	0.11	0.91	0.92
2018-S &P500-1step	0.42	0.91	0.88
2019-S &P500-1step	0.32	0.97	0.93
11.2019–10.2020-IBM-1step	0.26	0.80	0.80
11.2019–10.2020-KO-1step	0.01	0.75	0.77
11.2019–10.2020-MCD-1step	0.14	0.51	0.56
11.2019–10.2020-SBUX-1step	0.18	0.67	0.67
11.2019–10.2020-CADJPY-1step	0.07	0.46	0.48
11.2019–10.2020-CNYJPY-1step	0.66	0.78	0.75
11.2019–10.2020-USDCNY-1step	0.36	0.98	0.96
11.2019–10.2020-EURJP-1step	0.19	0.95	0.95
11.2019–10.2020-Djones-1step	0.30	0.56	0.55
11.2019–10.2020-S &P500-1step	0.25	0.59	0.58

The null hypothesis of the CW-test is that parsimonious and larger models have equal MSPE. The alternative is that the larger model has a smaller MSPE. The performances of GA-NoVaS and P-GA-NoVaS methods are calculated as we did in “Real-world data analysis” section, which are relative performances compared with the benchmark method (GARCH-direct method)

### MCS-test

Accompanied by the CW test, we utilize the Model Confidence Set (MCS)^11^ to determine a set of preferred models. The procedure for building the MCS is made up of a sequence of tests (henceforth MCS-test), where the null hypothesis of equal predictive ability (EPA) is not rejected at a specific confidence level. The advantage of the MCS test is that we can apply different loss functions, such as MSE and QLIKE, to compute the test statistics corresponding to different models and then select the best models. Moreover, we can rank the models in the MCS based on their prediction performance. Here, according to Eq. 8 of Bernardi and Catania (2018), we use the second test statistic $T_{max,M}$ to rank all models. To compare the different models, we propose three criteria: (1) Average Rank Order (ARO), which is the average of rank orders with respect to each model; (2) Confidence Set Rate (CSR), which is the relative frequency of each model belonging to the MCS; and (3) Best Model Rate (BMR), which is the relative frequency of each model ranking first. Similar criteria are defined in Amendola and Candila (2016). The three criteria are as follows:

27 $$\begin{aligned} ARO_i = \frac{1}{D}\sum _{d=1}^{D}r_{d,i}~;~CSR_i = \frac{1}{D}\sum _{d=1}^{D}I(M_{d,i}\subset {\hat{M}}^{*}_{d,1-\alpha })~;~BMR_i = \frac{1}{D}\sum _{d=1}^{D}FM_{d,i}, \end{aligned}$$

where $ARO_i$, $CSR_i$, and $BMR_i$ represent the ARO, CSR, and BMR for the *i*th interested model, respectively; *D* represents the number of datasets we apply for comparisons; $r_{d,i}$ stands for the rank order of the *i*th model on the *d*th dataset; $I(M_{d,i}\subset {\hat{M}}^{*}_{d,1-\alpha })$ is the indicator function, which is equal to 1 if the *i*th model belongs to the MCS ${\hat{M}}^{*}_{d,1-\alpha }$ for the *d*th dataset, otherwise it equals 0; $FM_{d,i}$ equals 1 if the *i*th ranks first for the *d*th dataset and equals 0 otherwise.

We run the MCS test on datasets that are applied to create Table 8. We take the confidence level of the MCS test to be 95$\%$ and adopt the MSE loss function to compute the test statistics. If one model is eliminated from the MCS, we set the corresponding rank order to 6. All the results are shown in Table 9. The P-GA-NoVaS method has the lowest ARO and the highest BMR. However, the GARCH-direct method does not win any first-rank title. In addition, we can see the “naive” GE-NoVaS method is dominated by other NoVaS methods. As indicated in Wu and Karmakar (2021), the P-GE-NoVaS method is superior to the GE-NoVaS method. Here, the claim is verified by the MCS-test again based on these three criteria. Interestingly, with the new transformation structure developed from the GARCH model, GA-NoVaS is competitive with P-GE-NoVaS even without applying the $\beta$-removing technique.

**Table 9:** The MCS-test rank order of all methods for 1-step ahead predictions on selected datasets

	GE-NoVaS	P-GE-NoVaS	GA-NoVaS	P-GA-NoVaS	GARCH-direct
2018-AAPL-1step	2	4	3	1	6
2019-AAPL-1step	4	1	3	2	5
2018-BAC-1step	4	2	3	1	6
2019-BAC-1step	6	1	4	2	3
2018-TSLA-1step	5	4	1	2	3
2019-TSLA-1step	4	1	5	2	3
2018-MCD-1step	4	2	3	1	5
2019-MCD-1step	3	1	4	2	6
2018-MSFT-1step	2	3	1	4	5
2019-MSFT-1step	5	2	3	1	4
2018-Djones-1step	4	3	2	1	5
2019-Djones-1step	4	1	3	2	5
2018-NASDAQ-1step	1	4	3	2	6
2019-NASDAQ-1step	5	2	3	1	4
2018-NYSE-1step	2	4	1	3	5
2019-NYSE-1step	4	1	2	3	5
2018-S &P500-1step	4	2	3	1	5
2019-S &P500-1step	4	1	3	2	5
11.2019–10.2020-IBM	3	4	2	1	6
11.2019–10.2020-KO	3	4	1	2	6
11.2019–10.2020-MCD	2	4	1	3	5
11.2019–10.2020-SBUX	3	4	2	1	6
11.2019–10.2020-CADJPY	1	4	2	3	5
11.2019–10.2020-CNYJPY	2	4	3	1	6
11.2019–10.2020-USDCNY	4	2	3	1	5
11.2019–10.2020-EURJPY	3	2	1	4	5
11.2019–10.2020-Djones	3	4	2	1	6
11.2019–10.2020-S &P500	3	4	2	1	6
ARO	3.357	2.679	2.464	**1.821**	5.071
CSR	0.964	**1.000**	**1.000**	**1.000**	0.642
BMR	0.071	0.250	0.214	**0.464**	0.000

The value 6 in Table 9 means the corresponding model is eliminated from the MCS for the specific dataset. We take the bold font to mark the best results according to three criteria

## Results and discussion

We conducted substantial simulation analyses to demonstrate the advantages of NoVaS methods for long-term forecasting and contrast our new methods with the existing NoVaS method. We compared as many as eight different simulation setups, and the highlighted benefits of the new methods are fairly uniform. In addition, we have provided a comprehensive real-data study to show that the advantages we are discussing in this paper not only stem from analyzing a particular dataset or even a particular type of data. We covered two different sizes of three different data types: (1) traditional stocks, (2) currency data, and (3) index data. Moreover, we covered three different lengths of the prediction horizon. After such a comprehensive range of experiments, we are confident that these methods will perform adequately well with any financial economic data from this wide range of forecasting exercises. Overall, the current state-of-the-art GE-NoVaS and our proposed new methods can avoid error accumulation problems, even when long-step ahead predictions are required. These methods outperform the GARCH(1,1) model in predicting either simulated or real-world data under different forecasting horizons.

In the future, we plan to explore the NoVaS method in various directions. Our new methods corroborate this and also open up avenues for exploring other specific transformation structures. In the financial market, stock data move together. Therefore, it would be interesting to see if one can make Model-free predictions for multiple time series directly. In certain areas, integer-valued time series have important applications. Thus, adjusting such Model-free predictions to handle count data is desirable. Moreover, with the advent of widely accessible high-frequency financial data, researchers have begun to investigate methods for digesting this abundant information within data, such as the heterogeneous autoregressive (HAR) model of Corsi (2009) and the GARCH model of Hansen et al. (2012). Application of the NoVaS prediction framework to high-frequency data could be a meaningful extension. In addition, the volatility forecasting returned by NoVaS methods could be considered as a meaningful feature that serves financial purposes, for example, bankruptcy prediction of small and medium-sized enterprises (SMEs) or complicated financial risk analysis; see related discussions from the work of Kou et al. (2014, 2021). There is also much scope in proving the statistical validity of such predictions. First, we hope to provide a rigorous and systematic way to compare the predictive accuracy of NoVaS-type and standard GARCH methods for time-aggregated forecasting. From a statistical inference point of view, one can also construct prediction intervals for these predictions using bootstrapping. Such prediction intervals are well sought in the econometrics literature, and some results on their asymptotic validity can be proved. We can also explore dividing the dataset into testing and training in an optimal manner and determine whether it can improve the performances of these methods. Beyond relying on Eq. (16) to measure different models, we can also consider proposing other measurements, such as the QLIKE-loss-based criterion. Simultaneously, the investigation of NoVaS methods for optimizing nonsymmetric loss could be a future work. Additionally, because determining the transformation function involves the optimization of unknown coefficients, designing a more efficient and precise algorithm may be a further direction for improving NoVaS-type methods.

## Conclusion

The NoVaS method is a non-parametric approach that can be used for many recursive time-series models. This study sheds new light on an attractive feature of the NoVaS method in the regime of conditional heteroscedastic models and then builds on new variants that can improve the state-of-the-art NoVaS methods designed for ARCH processes. Moreover, the newly proposed GA-NoVaS method has a more stable structure for handling volatile and short data than the already competent GE-NoVaS method. It can also bring about significant improvements when long-term prediction is desired. Additionally, although we reveal that parsimonious variants of GA-NoVaS and GE-NoVaS possess the same structure, the P-GA-NoVaS method is still more favorable because the corresponding region of the model parameter is more complete by design. In addition, the result from the CW test also indicates the possibility of achieving a good forecasting performance with the parsimonious version of the GA-NoVaS. In summary, the approach to building the NoVaS transformation using the GARCH(1,1) model is sensible and results in superior GA-NoVaS-type methods.

## Methods/experimental

In this study, we consider five methods: (1) the current NoVaS-type method, (2) GE-NoVaS and its parsimonious variant, (3) P-GE-NoVaS, (4) the newly proposed GA-NoVaS and P-GA-NoVaS, and (5) standard GARCH(1,1). To compare the performances of these methods with long-term time-aggregated predictions of volatility, we deployed simulations using eight GARCH-type models. We also selected comprehensive real-world datasets that cover traditional stock, currency, and index data. The prediction procedure and evaluation metrics are explained in “Long-term forecasting evaluation metric” section. Moreover, we substantiated the superiority of our methods using the CW and MCS tests. These tests are described in “Comparison of predictive accuracy: statistical tests” section. All data analyses are parallelly computed in the *R*-studio.

## Figures and Tables

**Fig. 2:**
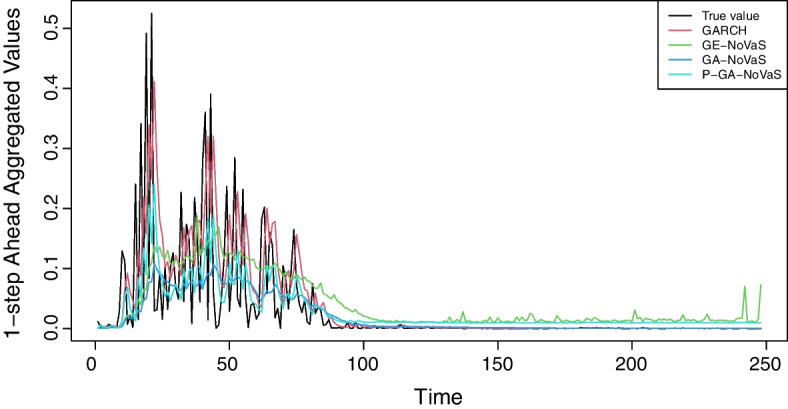
The curves of different methods’ 1-step ahead aggregated predictions on 500 simulated Model 8 data and actual values

**Fig. 3:**
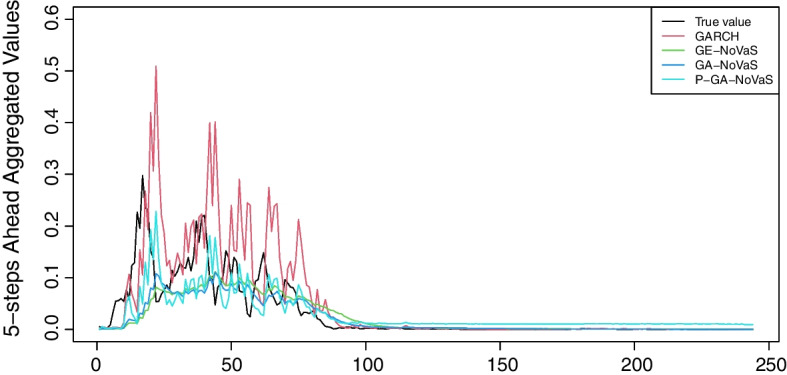
The curves of different methods’ 5-steps ahead aggregated predictions on 500 simulated Model 8 data and actual values

**Fig. 4:**
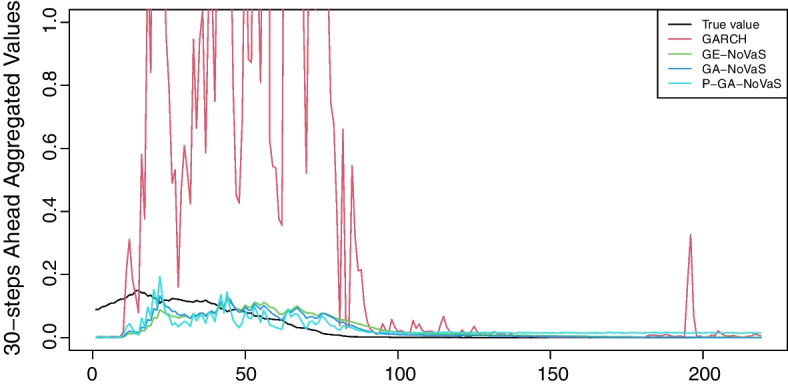
The curves of different methods’ 30-steps ahead aggregated predictions on 500 simulated Model 8 data and actual values

**Fig. 5:**
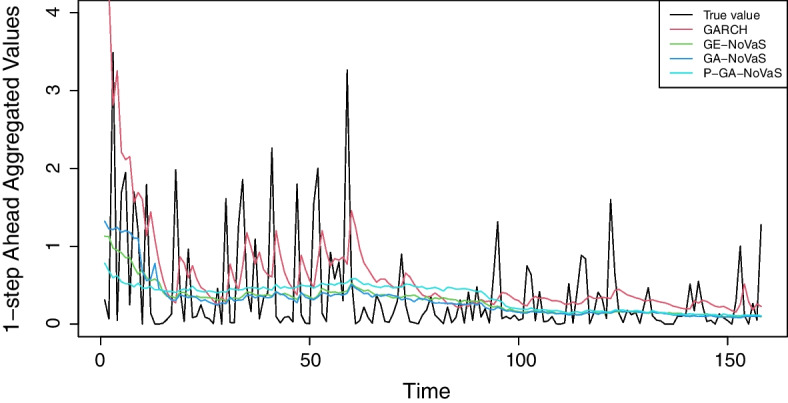
The curves of different methods’ 1-step ahead aggregated predictions on volatile CADJPY data and actual values

**Fig. 6:**
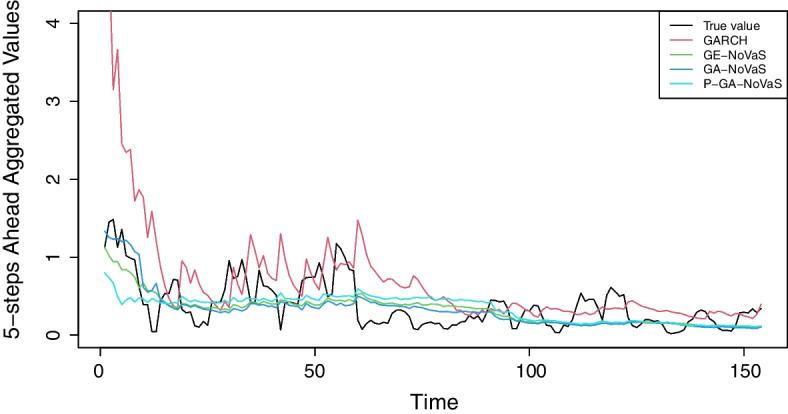
The curves of different methods’ 5-steps ahead aggregated predictions on volatile CADJPY data and actual values

**Fig. 7:**
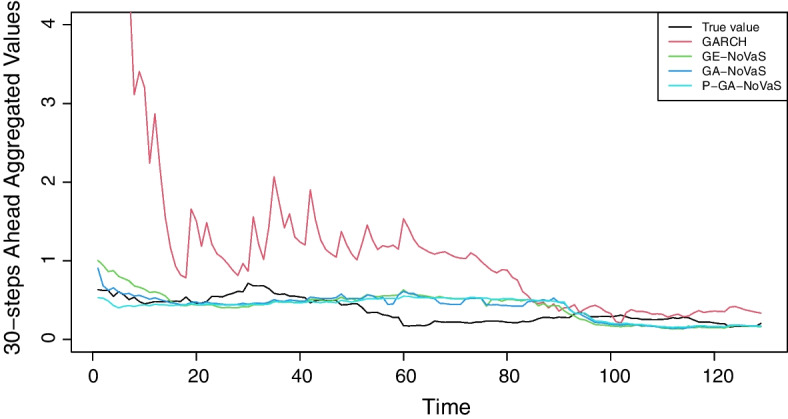
The curves of different methods’ 30-steps ahead aggregated predictions on volatile CADJPY data and actual values

## Data Availability

We have collected all data presented here from www.investing.com manually. Then, we transform the closing price data to financial log-returns based on Eq 4.1 in the manuscript.

## Appendix

### Appendix 1: The deduction of $H_{T}$ and $H_{T}^{-1}$ corresponding with the GA-NoVaS method

Based on Eq. (17), we first find out expressions of $\sigma _{t-1}^2,\sigma _{t-2}^2,\ldots$ as follow:

28 $$\begin{aligned} \begin{aligned} \sigma _{t-1}^2&= a + a_1Y_{t-2}^2 + b_1\sigma _{t-2}^2,\\ \sigma _{t-2}^2&= a + a_1Y_{t-3}^2 + b_1\sigma _{t-3}^2,\\ \vdots&\end{aligned} \end{aligned}$$

Plug all components in Eq. (28) into Eq. (17), one equation sequence can be gotten:

29 $$\begin{aligned} \begin{aligned} Y_t&= W_t\sqrt{a + a_1Y_{t-1}^2 + b_1\sigma _{t-1}^2}\\&= W_t\sqrt{a + a_1Y_{t-1}^2 + b_1(a + a_1Y_{t-2}^2 + b_1\sigma _{t-2}^2)}\\&= W_t\sqrt{a + a_1Y_{t-1}^2 + b_1a + b_1a_1Y_{t-2}^2 + b_1^2(a + a_1Y_{t-3}^2 + b_1\sigma _{t-3}^2)}\\&\vdots \end{aligned} \end{aligned}$$

Iterating the process in Eq. (29), with the requirement of $a_1+b_1<1$ for the stationarity, the limiting form of $Y_t$ can be written as Eq. (30):

30 $$\begin{aligned} Y_t =W_t\sqrt{ \sum _{i = 1}^{\infty }a_1b_1^{i-1}Y_{t-i}^2 + \sum _{j=0}^{\infty }ab_1^j} = W_t\sqrt{ \sum _{i = 1}^{\infty }a_1b_1^{i-1}Y_{t-i}^2 + \frac{a}{1-b_1}}. \end{aligned}$$

We can rewrite Eq. (30) to get a potential function $H_T$ which is corresponding to the GA-NoVaS method:

31 $$\begin{aligned} W_t = \frac{Y_t}{\sqrt{ \sum _{i = 1}^{\infty }a_1b_1^{i-1}Y_{t-i}^2 + \frac{a}{1-b_1}}}. \end{aligned}$$

Recall the adjustment taken in the existing GE-NoVaS method, the total difference between Eq. (4,5) can be seen as the term *a* being replaced by $\alpha s_{t-1}^2 + \beta Y_t^2$. Apply this same adjustment on Eq. (31), then this equation will be changed to the form as follows:

32 $$\begin{aligned} W_t = \frac{Y_t}{\sqrt{ \frac{\beta Y_t^2 + \alpha s_{t-1}^2}{1-b_1}+ \sum _{i = 1}^{\infty }a_1b_1^{i-1}Y_{t-i}^2 }} = \frac{Y_t}{\sqrt{ \frac{\beta Y_t^2}{1-b_1}+ \frac{\alpha s_{t-1}^2}{1-b_1} + \sum _{i = 1}^{\infty }a_1b_1^{i-1}Y_{t-i}^2 }}. \end{aligned}$$

In Eq. (32), since $\alpha /(1-b_1)$ is also required to take a small positive value, this term can be seen as a $\tilde{\alpha }$ ($\tilde{\alpha } \ge 0$) which is equivalent with $\alpha$ in the existing GE-NoVaS method. Thus, we can simplify $\alpha s_{t-1}^2/(1-b_1)$ to $\tilde{\alpha } s_{t-1}^2$. For keeping the same notation style with the GE-NoVaS method, we use $\alpha s_{t-1}^2$ to represent $\alpha s_{t-1}^2/(1-b_1)$. Then Eq. (32) can be represented as:

33 $$\begin{aligned} W_t = \frac{Y_t}{\sqrt{ \frac{\beta Y_t^2}{1-b_1}+ \alpha s_{t-1}^2 + \sum _{i = 1}^{\infty }a_1b_1^{i-1}Y_{t-i}^2 }}. \end{aligned}$$

For getting a qualified GA-NoVaS transformation, we still need to make the transformation function Eq. (33) satisfy the requirement of the Model-free Prediction Principle. Recall that in the existing GE-NoVaS method, $\alpha + \beta + \sum _{i=1}^pa_i$ in Eq. (5) is restricted to be 1 for meeting the requirement of variance-stabilizing and the optimal combination of $\alpha ,\beta , a_1,\ldots ,a_p$ is selected to make the empirical distribution of $\{W_t\}$ as close to the standard normal distribution as possible (i.e., minimizing $|KURT(W_t)-3|$). Similarly, for getting a qualified $H_T$ from Eq. (33), we require:

34 $$\begin{aligned} \frac{\beta }{1-b_1} +\alpha + \sum _{i=1}^{\infty }a_1b_1^{i-1} = 1. \end{aligned}$$

Under this requirement, since $a_1$ and $b_1$ are both less than 1, $a_1b_1^{i-1}$ will converge to 0 as *i* converges to $\infty$, i.e., $a_1b_1^{i-1}$ is neglectable when *i* takes large values. So it is reasonable to replace $\sum _{i=1}^{\infty }a_1b_1^{i-1}$ in Eq. (34) by $\sum _{i=1}^{q}a_1b_1^{i-1}$, where *q* takes a large value. Then, Eq. (35) is obtained:

35 $$\begin{aligned} W_t = \frac{Y_t}{\sqrt{ \frac{\beta Y_t^2}{1-b_1}+ \alpha s_{t-1}^2 + \sum _{i = 1}^{q}a_1b_1^{i-1}Y_{t-i}^2 }}~;~\text {for}~ t=q+1,\ldots ,T. \end{aligned}$$

Now, we take Eq. (35) as a potential function $H_T$. Then, the requirement of variance-stabilizing is changed to:

36 $$\begin{aligned} \frac{\beta }{1-b_1} +\alpha + \sum _{i=1}^{q}a_1b_1^{i-1} = 1. \end{aligned}$$

Akin to Eq. (7), we scale $\{\frac{\beta }{1-b_1},a_1,a_1b_1$ $,a_1b_1^{2},$ $\ldots ,a_1b_1^{q-1} \}$ of Eq. (36) by timing a scalar $\frac{1-\alpha }{\frac{\beta }{1-b_1} + \sum _{i=1}^{q}a_1b_1^{i-1}}$, and then search optimal coefficients. For presenting Eq. (35) with scaling coefficients in a concise form, we use $\{c_0,c_1,\ldots ,c_q\}$ to represent $\{\frac{\beta }{1-b_1},a_1,a_1b_1$ $,a_1b_1^{2},$ $\ldots ,a_1b_1^{q-1} \}$ after scaling, which implies that we can rewrite Eq. (35) as:

37 $$\begin{aligned} W_t = 
\frac{Y_t}{\sqrt{ c_0Y_t^2+ \alpha s_{t-1}^2 + \sum _{i = 1}^{q}c_iY_{t-i}^2 }}~;~\text {for}~ t=q+1,\ldots ,T. \end{aligned}$$

Furthermore, for achieving the aim of normalizing, based on Eq. (37), we still fix $\alpha$ to be one specific value from $\{0.1,0.2,\ldots ,0.8\}$, and then search the optimal combination of $\beta ,a_1,b_1$ from three grids of possible values of $\beta ,a_1,b_1$ to minimize $|KURT(W_t)-3|$. After getting a qualified $H_T$, $H_T^{-1}$ can be outlined immediately:

38 $$\begin{aligned} Y_t = \sqrt{\frac{W_t^2}{1-c_0W_t^2}(\alpha s_{t-1}^2+\sum _{i=1}^qc_iY_{t-i}^2)}~;~\text {for}~ t=q+1,\ldots ,T. \end{aligned}$$

Based on Eq. (38), for example, $Y_{T+1}$ can be expressed as the equation follows:

39 $$\begin{aligned} Y_{T+1} = \sqrt{\frac{W_{T+1}^2}{1-c_0W_{T+1}^2}(\alpha s_{T}^2+\sum _{i=1}^qc_iY_{T+1-i}^2)}. \end{aligned}$$

### Appendix 2: The comparison of parsimonious GE-NoVaS and GA-NoVaS methods

We asserted that the P-GA-NoVaS method works better than the P-GE-NoVaS in Eq. (3.3). Although these two parsimonious variants of GE-NoVaS and GA-NoVaS have the same structure, we showed that the regions of their parameters are different. The P-GA-NoVaS method has a wider parameter space, and this property implies that it is a more complete technique. For substantiating this idea, we compare the forecasting performance of these two parsimonious methods and present results in Table 10. We use the bold values mark cases where one of these two methods is at least 10% better than the other one based on the relative prediction performance. We can find most cases are accompanied by very small relative values, which indicates that these two methods stand almost the same performance and is in harmony with the fact that they share the same structure. However, we can find there are 21 cases where the P-GA-NoVaS method works at least 10$\%$ better than the P-GE-NoVaS method. On the other hand, there are only 8 cases where the P-GE-NoVaS method shows significantly better results. We shall notice that the P-GA-NoVaS method is optimized by determining parameters from several grids of values. Therefore, we can imagine the performance of this method will further increase if more refined grids are used.

**Table 10:** Comparisons of P-GE-NoVaS and P-GA-NoVaS methods

	P-GE	P-GA	Relative value
*500 simulated data*			
M1-1step	0.85483	0.84138	0.02
M1-5steps	0.40510	0.40137	0.01
M1-30steps	0.03293	0.03290	0.00
M2-1step	0.98431	0.99658	0.01
M2-5steps	0.90156	0.91980	0.02
M2-30steps	0.49761	0.48396	0.03
M3-1step	0.99360	0.98407	0.01
M3-5steps	0.94456	0.94073	0.00
M3-30steps	0.92128	0.90855	0.01
M4-1step	0.97484	0.99640	0.02
M4-5steps	0.90862	0.95338	0.05
M4-30steps	0.68266	0.67594	0.01
M5-1step	0.96936	0.97151	0.00
M5-5steps	0.82810	0.82747	0.00
M5-30steps	0.53026	**0.47311**	0.11
M6-1step	0.90196	0.93509	0.04
M6-5steps	0.75922	0.80311	0.05
M6-30steps	0.40790	0.41112	0.01
M7-1step	0.96081	0.95932	0.00
M7-5steps	0.84889	0.85127	0.00
M7-30steps	0.69698	0.71391	0.02
M8-1step	0.92557	0.93452	0.01
M8-5steps	0.92549	0.93178	0.01
M8-30steps	0.33834	0.33853	0.00
*250 simulated data*			
M1-1step	0.83168	0.83034	0.00
M1-5steps	0.43772	0.43247	0.01
M1-30steps	0.22659	0.23035	0.02
M2-1step	0.88781	0.87614	0.01
M2-5steps	0.52872	0.51712	0.02
M2-30steps	0.73604	0.75251	0.02
M3-1step	0.94635	0.93693	0.01
M3-5steps	0.96361	0.99977	0.04
M3-30steps	0.98872	0.99818	0.01
M4-1step	0.92829	0.92811	0.00
M4-5steps	0.67482	0.67894	0.01
M4-30steps	**0.71003**	0.81115	0.12
M5-1step	0.78087	0.79075	0.01
M5-5steps	0.34396	0.35155	0.02
M5-30steps	0.00201	0.00194	0.03
M6-1step	0.94661	0.93863	0.01
M6-5steps	0.84719	0.85851	0.01
M6-30steps	0.70301	0.70420	0.00
M7-1step	**0.73553**	0.83216	0.12
M7-5steps	0.46618	0.48020	0.03
M7-30steps	0.06479	0.06507	0.00
M8-1step	0.76586	0.83340	0.08
M8-5steps	**0.38107**	0.45665	0.17
M8-30steps	0.00918	0.00983	0.07
*Previous 2-year real-world data*			
AAPL-1step	0.98261	0.97836	0.00
AAPL-5steps	1.00090	0.96999	0.03
AAPL-30steps	0.97326	0.96174	0.01
BAC-1step	1.00416	1.02780	0.02
BAC-5steps	0.97542	0.99399	0.02
BAC-30steps	0.99533	1.00363	0.01
MSFT-1step	0.97376	0.96185	0.01
MSFT-5steps	0.96601	0.95271	0.01
MSFT-30steps	0.91046	0.88515	0.03
MCD-1step	0.98873	0.99005	0.00
MCD-5steps	0.98059	0.97777	0.00
MCD-30steps	0.99512	0.99418	0.00
1-year stock data			
MCD18-1step	0.95332	0.94412	0.01
MCD18-5steps	0.88773	0.88151	0.01
MCD18-30steps	0.61097	0.61153	0.00
MCD19-1step	0.93141	0.94559	0.01
MCD19-5steps	0.90061	0.90602	0.01
MCD19-30steps	0.80805	0.77976	0.04
AAPL18-1step	0.96311	0.89283	0.07
AAPL18-5steps	0.85114	**0.71233**	0.16
AAPL18-30steps	0.35731	0.37081	0.04
AAPL19-1step	0.80948	0.81872	0.01
AAPL19-5steps	0.68191	0.68792	0.01
AAPL19-30steps	0.73823	0.72892	0.01
BAC18-1step	0.93031	0.92619	0.00
BAC18-5steps	0.86387	**0.72512**	0.16
BAC18-30steps	0.81429	0.87630	0.07
BAC19-1step	0.97757	0.98605	0.01
BAC19-5steps	0.89571	0.95436	0.06
BAC19-30steps	1.01175	1.03482	0.02
MSFT18-1step	0.94507	0.95921	0.01
MSFT18-5steps	0.76646	0.74237	0.03
MSFT18-30steps	0.68741	0.64717	0.06
MSFT19-1step	0.98469	0.95347	0.03
MSFT19-5steps	1.02387	0.95417	0.07
MSFT19-30steps	0.97585	0.98476	0.01
Tesla18-1step	0.95885	**0.86238**	0.10
Tesla18-5steps	1.02036	1.01560	0.00
Tesla18-30steps	1.22256	1.25005	0.02
Tesla19-1step	0.98646	0.98955	0.00
Tesla19-5steps	0.97523	0.96107	0.01
Tesla19-30steps	0.87158	0.84366	0.03
Bitcoin18-1step	0.99967	0.97734	0.02
Bitcoin18-5steps	1.02008	0.93826	0.08
Bitcoin18-30steps	0.95020	0.91364	0.04
Bitcoin19-1step	0.86795	0.87256	0.01
Bitcoin19-5steps	0.55620	0.56789	0.02
Bitcoin19-30steps	0.00624	0.00631	0.01
*1-year index data*			
NASDAQ18-1step	0.94837	0.92421	0.03
NASDAQ18-5steps	0.83585	0.78823	0.06
NASDAQ18-30steps	0.74660	**0.67389**	0.10
NASDAQ19-1step	0.93558	0.93843	0.00
NASDAQ19-5steps	0.84459	0.84051	0.00
NASDAQ19-30steps	0.58924	0.59218	0.00
NYSE18-1step	0.95793	0.96619	0.01
NYSE18-5steps	0.87919	**0.75822**	0.14
NYSE18-30steps	0.79466	**0.61830**	0.22
NYSE19-1step	0.90407	0.92193	0.02
NYSE19-5steps	0.69822	0.71038	0.02
NYSE19-30steps	0.18173	0.18116	0.00
Smallcap18-1step	0.91299	0.91101	0.00
Smallcap18-5steps	0.73541	0.73223	0.00
Samllcap18-30steps	0.48461	0.46312	0.04
Smallcap19-1step	0.98731	0.98482	0.00
Smallcap19-5steps	0.87700	0.85490	0.03
Samllcap19-30steps	0.88825	0.90360	0.02
Djones18-1step	0.84931	0.91192	0.07
Djones18-5steps	0.86017	**0.76226**	0.11
Djones18-30steps	0.66418	**0.56854**	0.14
Djones19-1step	0.96365	0.96977	0.01
Djones19-5steps	0.89542	0.91238	0.02
Djones19-30steps	0.80304	0.81803	0.02
S &P500-18-1step	0.90469	0.88405	0.02
S &P500-18-5steps	0.90544	**0.75646**	0.16
S &P500-18-30steps	0.83210	**0.72104**	0.13
S &P500-19-1step	0.92183	0.93162	0.01
S &P500-19-5steps	0.75579	0.77434	0.02
S &P500-19-30steps	0.29796	0.30127	0.01
BSE18-1step	0.94676	0.92568	0.02
BSE18-5steps	0.82886	0.84408	0.02
BSE18-30steps	0.44818	0.44092	0.02
BSE19-1step	0.67694	0.66667	0.02
BSE19-5steps	0.23665	0.23603	0.00
BSE19-30steps	0.02890	0.02888	0.00
BIST18-1step	0.92271	0.94138	0.02
BIST18-5steps	0.80837	0.81417	0.01
BIST18-30steps	1.09665	1.09900	0.00
BIST19-1step	0.95467	0.98234	0.03
BIST19-5steps	0.82898	0.85370	0.03
BIST19-30steps	0.88511	0.85743	0.03
*Volatile 1-year stock data*			
NKE-1step	0.65295	0.65594	0.00
NKE-5steps	0.22550	0.22226	0.01
NKE-30steps	0.00337	0.00340	0.01
AMZN-1step	0.90200	0.90487	0.00
AMZN-5steps	0.71789	0.72850	0.01
AMZN-30steps	0.53460	0.53310	0.00
IBM-1step	0.80744	0.79509	0.02
IBM-5steps	0.40743	0.38413	0.06
IBM-30steps	0.00918	0.00879	0.04
MSFT-1step	0.84502	0.81582	0.03
MSFT-5steps	0.37528	0.38022	0.01
MSFT-30steps	0.00732	0.00784	0.07
SBUX-1step	0.69943	0.66743	0.05
SBUX-5steps	0.30528	**0.26856**	0.12
SBUX-30steps	0.00289	**0.00236**	0.18
KO-1step	0.81309	0.77035	0.05
KO-5steps	0.39679	**0.33405**	0.16
KO-30steps	0.01963	**0.01582**	0.19
MCD-1step	0.58018	0.56414	0.03
MCD-5steps	0.17887	0.17439	0.03
MCD-30steps	0.00001	0.00001	0.02
Tesla-1step	0.89253	0.88782	0.01
Tesla-5steps	0.66177	0.66937	0.01
Tesla-30steps	0.22460	0.22395	0.00
Bitcoin-1step	0.36346	0.36326	0.00
Bitcoin-5steps	0.01321	0.01322	0.00
Bitcoin-30steps	0.00000	0.00000	0.00
*Volatile 1-year currency data*			
CADJPY-1step	0.48712	0.48367	0.01
CADJPY-5steps	0.13549	0.14376	0.06
CADJPY-30steps	**0.00394**	0.00482	0.18
EURJPY-1step	0.94206	0.95133	0.01
EURJPY-5steps	0.76727	0.75636	0.01
EURJPY-30steps	**0.15350**	0.18189	0.16
USDCNY-1step	0.96766	0.95757	0.01
USDCNY-5steps	0.86364	0.83523	0.03
USDCNY-30steps	0.60121	0.60131	0.00
GBPJPY-1step	0.86553	0.87160	0.01
GBPJPY-5steps	0.49208	0.48842	0.01
GBPJPY-30steps	0.17246	0.17262	0.00
USDINR-1step	0.98975	0.93140	0.06
USDINR-5steps	0.81103	0.75693	0.07
USDINR-30steps	0.17966	**0.11380**	0.37
CNYJPY-1step	0.79877	0.74586	0.07
CNYJPY-5steps	0.40569	**0.34839**	0.14
CNYJPY-30steps	0.06270	**0.05444**	0.13
*Volatile 1-year index data*			
S &P500-1step	0.92349	0.92854	0.01
S &P500-5steps	0.75183	0.77060	0.02
S &P500-30steps	0.29793	0.30115	0.01
NASDAQ-1step	0.75350	0.77753	0.03
NASDAQ-5steps	0.33519	0.36428	0.08
NASDAQ-30steps	**0.00599**	0.00696	0.14
NYSE-1step	0.57174	0.54598	0.05
NYSE-5steps	0.10182	**0.07798**	0.23
NYSE-30steps	0.00001	0.00001	0.01
Smallcap-1step	0.60931	0.57773	0.05
Smallcap-5steps	0.10337	0.09628	0.07
Smallcap-30steps	0.00006	**0.00004**	0.39
BSE-1step	0.39745	0.39851	0.00
BSE-5steps	0.04109	0.04170	0.01
BSE-30steps	0.00002	0.00002	0.05
DAX-1step	0.64727	0.66097	0.02
DAX-5steps	0.10356	0.11085	0.07
DAX-30steps	0.00007	0.00008	0.06
USDX future-1step	0.99640	1.03693	0.04
USDX future-5steps	0.54834	0.51997	0.05
USDX future-30steps	0.10278	0.10063	0.02
Bovespa-1step	0.57558	0.60656	0.05
Bovespa-5steps	**0.07447**	0.09395	0.21
Bovespa-30steps	**2.04E**−**6**	3.19E−6	0.36
Djones-1step	0.57550	0.54422	0.05
Djones-5steps	0.11554	**0.08698**	0.25
Djones-30steps	**0.00001**	0.00003	0.53
BIST-1step	0.90832	0.92418	0.02
BIST-5steps	0.47480	0.49279	0.04
BIST-30steps	0.05550	0.05671	0.02

### Appendix 3: The detailed analysis of simulation results with Models 6 and 8

From Table 1, a remarkable case is the 500-size simulation of Model 8, where NoVaS-type methods achieve incredible victory compared to the GARCH-direct method. Here, we want to present plots of different methods’ predictions and true values in the same figure to compare them directly in an absolute sense. Different methods’ performance on 1-step, 5-steps and 30-steps ahead aggregated predictions of 500 simulated Model 8 data are figured in Figs. 2, 3 and 4, respectively. It is clear that the GARCH-direct method is quite unstable for long-term (5-steps and 30-steps ahead) aggregated predictions. On the other hand, NoVaS-type methods can capture the basic trend of simulated data for both short- and long-term aggregated predictions. In addition, we further investigate whether the NoVaS method is robust against the model misspecification. Thus, we estimate a GJR-GARCH(1,1) model and then do predictions. Setting the benchmark method as GJR-GARCH(1,1) model, we tabulated the comparison result among various NoVaS, GARCH(1,1) and GJR-GARCH(1,1) methods in Table 11. For both short- and long-term forecasting, NoVaS-type methods stand for generally acceptable performance. Particularly, the GA-NoVaS method works even better than the GJR-GARCH(1,1) model on 30-steps ahead aggregated predictions. Similarly, we apply the above procedure to Model 6 which is an Exponential GARCH(1,1) (EXP-GARCH(1,1)) model. We also estimate an Exponential GARCH(1,1) model to do predictions and set it as the benchmark. The comparison of different methods is tabulated in Table 12. The results show the P-GA-NoVaS method is the best one for all three prediction horizons, which highlights the superior ability of NoVaS methods on forecasting short data.

**Table 11:** Comparison results of NoVaS-type, GARCH(1,1) and GJR-GARCH(1,1) methods on forecasting 500 size simulated Model 8 data

500 size	GE-NoVaS	GA-NoVaS	P-GA-NoVaS	GARCH-direct	GJR-GARCH
M8-1step	1.08923	1.10182	1.11782	1.36494	1.00000
M8-5steps	1.34595	1.31927	1.37408	3.85497	1.00000
M8-30steps	1.19125	0.97943	1.06189	1125.00581	1.00000

The benchmark is the GJR-GARCH(1,1) method, so numerical values in the table corresponding to this method are 1. Other numerical values are relative performance compared to the GJR-GARCH(1,1) method. This table is also based on the average result of 5 replications

**Table 12:** Comparison results of NoVaS-type, GARCH(1,1) and Exponential-GARCH(1,1) methods on forecasting 500 size simulated Model 6 data

500 size	GE-NoVaS	GA-NoVaS	P-GA-NoVaS	GARCH-direct	EXP-GARCH
M8-1step	1.03388	1.02927	0.98002	1.00477	1.00000
M8-5steps	1.00765	1.01167	0.91506	0.98363	1.00000
M8-30steps	1.07155	1.06845	0.92544	1.03187	1.00000

The benchmark is the EXP-GARCH(1,1) method, so numerical values in the table corresponding to this method are 1. Other numerical values are relative performance compared to the EXP-GARCH(1,1) method. This table is also based on the average result of 5 replications

### Appendix 4: Comparison plots of the forecasting on the volatile CADJPY data

Based on the empirical analyses, a remarkable result is the forecasting of the volatile CADJPY case from Table 6, where the GA-NoVaS method dominates other models. As we did in “Appendix 3”, we present comparison plots of the 1-step, 5-steps and 30-steps ahead aggregated forecast on volatile CADJPY data in Figs. 5, 6 and 7, respectively. Still, the GARCH-direct method is quite off the actual curve for long-term predictions. On the other hand, NoVaS-type methods are obviously more stable in an absolute sense.

## Abbreviations

NoVaS: Normalizing and variance stabilizing
FITVGARCH: Fractionally integrated time-varying GARCH
POOS: Seudo-out of sample
GE-NoVaS: Generalized Exponential NoVaS
GA-NoVaS: GARCH-NoVaS
P-GE-NoVaS: Parsimonious GE-NoVaS
P-GA-NoVaS: Parsimonious GA-NoVaS
MCS: Model confidence set
EPA: Equal predictive ability
ARO: Average rank order
CSR: Confidence set rate
BMR: Best model rate
HAR: Heterogeneous autoregressive model
EXP-GARCH(1,1): Exponential GARCH(1,1)

## Footnotes

1 Squared log-returns are unbiased, but a very noisy measure of volatility pointed out by Andersen and Bollerslev (1998). Additionally, Awartani and Corradi (2005) showed that using squared log-returns as a proxy for volatility can render a correct ranking of different GARCH models in terms of a quadratic loss function.

2 The POOS forecasting analysis means using data up to and including current time to predict future values.

3 The “Model-free” in this context means we do not rely on a statistical model to do predictions. Although a transformation function needs to be estimated, it is just a “bridge” which connects original and transformed distributions.

4 If $\{W_t\}_{t=p+1}^{T}$ is correlated, some additional manipulations need to be done, more details can be found in Politis (2015).

5 More details about this minimizing process can be found in Politis (2015).

6 It is possible to refine this grid to get a better transformation. However, the computation burden will also increase.

7 The reason for utilizing a trimmed standard normal distribution is transformed $\{W_t\}_{t=p+1}^{T}$ are between $-1/\sqrt{\beta }$ and $1/\sqrt{\beta }$ from Eq. (5).

8 When *c* is large, $a_i \approx 0$ for all $i > 0$. It is hard to make the kurtosis of transformed series be 3.

9 Due to the sliding-window prediction property, we only repeat each simulation 5 times and present average results.

10 See Clark and West (2007) for the theoretical details of this test, explaining that these details are not within the scope of this study.

11 See Hansen et al. (2011) for theoretical details and the practical directions can be found in Bernardi and Catania (2018).
